# 2D MXene Nanomaterials as Electrocatalysts for Hydrogen Evolution Reaction (HER): A Review

**DOI:** 10.3390/mi13091499

**Published:** 2022-09-09

**Authors:** Shaik Gouse Peera, Ravindranadh Koutavarapu, Liu Chao, Lakhveer Singh, Govindhasamy Murugadoss, Gaddam Rajeshkhanna

**Affiliations:** 1 Department of Environmental Science, Keimyung University, Dalseo-gu, Daegu 42601, Korea; 2 Department of Robotics Engineering, College of Mechanical and IT Engineering, Yeungnam University, Gyeongsan 38541, Korea; 3 Engineering Research Center for Hydrogen Energy Materials and Devices, Faculty of Materials Metallurgy and Chemistry, Jiangxi University of Science and Technology, Ganzhou 341000, China; 4 Department of Chemistry, Sardar Patel University, Mandi 175001, Himachal Pradesh, India; 5 Department of Civil Engineering, Center for Research & Development, Chandigarh University, Mohali 140413, Punjab, India; 6 Centre for Nanoscience and Nanotechnology, Sathyabama Institute of Science and Technology, Chennai 600119, Tamilnadu, India; 7 Department of Chemistry, National Institute of Technology Warangal, Warangal 506004, Telangana, India

**Keywords:** 2D MXene, Ti_3_C_2_T_x_, water splitting, hydrogen evolution reaction, Tafel slope

## Abstract

MXenes, a novel family of 2D transition metal carbide, nitride and carbonitride materials, have been gaining tremendous interest in recent days as potential electrocatalysts for various electrochemical reactions, including hydrogen evolution reaction (HER). MXenes are characterized by their etchable metal layers, excellent structural stability, versatility for heteroatoms doping, excellent electronic conductivity, unique surface functional groups and admirable surface area, suitable for the role of electrocatalyst/support in electrochemical reactions, such as HER. In this review article, we summarized recent developments in MXene-based electrocatalysts synthesis and HER performance in terms of the theoretical and experimental point of view. We systematically evaluated the superiority of the MXene-based catalysts over traditional Pt/C catalysts in terms of HER kinetics, Tafel slope, overpotential and stability, both in acidic and alkaline electrolytic environments. We also pointed out the motives behind the electro catalytic enhancements, the effect of synthesis conditions, heteroatom doping, the effect of surface terminations on the electrocatalytic active sites of various MXenes families. At the end, various possible approaches were recommended for a deeper understanding of the active sites and catalytic improvement of MXenes catalysts for HER.

## 1. Introduction

**Figure 1:**
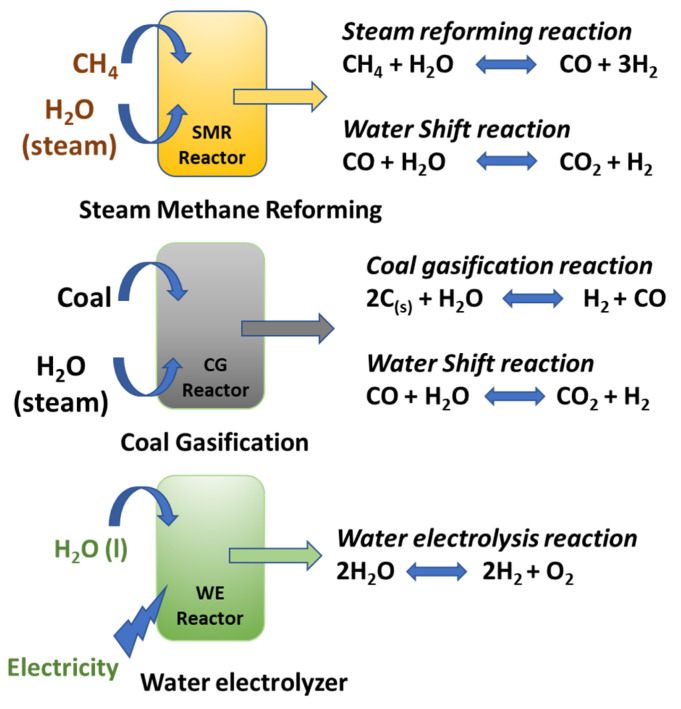
The three major pathways for industrial hydrogen production.

Increased global demand for energy is expected to be doubled by 2035 due to increased economic advancements, both in developed and developing countries [1]. At present, most of the energy demands are fulfilled by nonrenewable energy sources, such as coal, fossil fuels and natural gas. However, their usage in the near future is going to be very limited due to increased consumption, scarcity and, most importantly, the emission of greenhouse gases that have a tremendous impact on global warming [2]. Therefore, it has become a serious and urgent issue to identify alternative, renewable energy sources than can replace/limit the use of the traditional nonrenewable energy supplies for the domestic, industrial and especially transportation sector [3]. Owing to its high gravimetric energy density (140 MJ/kg), hydrogen (H_2_) is considered the best energy source for transportation applications. For example, energy conversion fuel cell uses gaseous H_2_ as the fuel source [4]. In addition, gaseous H_2_ is also used in a wide variety of industries and chemical reactions, such as NH_3_ production and the fertilizers manufacturing sector. To meet such a high demand, in general, gaseous H_2_ is produced from steam reforming and coal gasification technologies [5]. However, the most popular steam reforming and coal gasification technologies cannot solve the problem of CO_2_ emission. In addition, these technologies also use high temperature for H_2_ production, selective catalysts, significant amount of CO_2_ along with a small quantity of impurities, such as CO (Figure 1). By no means can steam reforming and coal gasification technologies be a long-term solution for gaseous H_2_ production. In addition, they aggravate the rapid depletion of fossil fuels and contribute to elevated global warming issues [6]. Therefore, the search for alternative and green energy technology for gaseous H_2_ production remains a challenging and interesting task for the scientific community.

As an alternative to the traditional technologies, H_2_ production by electrochemical water splitting/water electrolysis has been gaining tremendous interest recently [7]. Electrochemical water splitting into O_2_ and gaseous H_2_ can be the best source of H_2_ energy, as it is a carbon-free technology, unlike steam reforming and coal gasification techniques. Further, water electrolysis can be performed by a variety of renewable energy resources, such as solar, wind, etc. [8]. Water electrolysis is performed by applying external voltage, which enables the decomposition of water into O_2_ and H_2_ gases. The O_2_ gas is produced at the anodes, whereas the H_2_ gas is produced at the cathodes [9]. Put simply, H_2_O oxidation occurs at the anodes, which leads to O_2_ evolution, referred to as an oxygen evolution reaction (OER), and H_2_O reduction occurs at the cathodes, which leads to H_2_ evolution, referred to as a hydrogen evolution reaction (HER). Water oxidation takes place both in acidic and alkaline electrolytes, and their respective reactions are given below.

Water electrolysis in acidic medium

Anode: 2H_2_O (l) → O_2_ (g) + 4H^+^ + 4e^−^ E° = 1.23 V vs. SHE

Cathode: 4H^+^ + 4e^−^ → 2H_2_ (g) E° = 0 V vs. SHE

Overall reaction: 2H_2_O (l) → 2H_2_ (g) +O_2_ (g) ΔE° = 1.23 V,

ΔG° = +237.2 kJ H_2_ mol^−1^

Water electrolysis in alkaline medium

Anode: 4OH^−^ → O_2_(g) + 2H_2_O + 4e^−^ E° = 0.40 V vs. SHE

Cathode: 4H_2_O + 4e^−^ → 2H_2_(g) +4 OH^−^ E° = −0.83 V vs. SHE

Overall reaction: 2H_2_O (l) → 2H_2_ (g) + O_2_ (g) ΔE° = 1.23 V,

ΔG° = +237.2 kJ H_2_ mol^−1^

From the above chemical reactions, it is to be noted that the ΔG° is positive (endothermic reaction) for water electrolysis reactions, irrespective of the electrolyte media. Therefore, the surplus external potential, known as the overpotential (η), greater than the thermodynamic potential (1.23 V vs. Reversible hydrogen electrode (RHE)) needs to be applied in order to achieve water electrolysis. In addition to the applied overpotential, a suitable, active and durable electrocatalyst is also essential in order to reduce the η, both at the anodes and the cathodes of the electrolyzer cell. Traditionally, various noble metal catalysts, such as Pt, Pd, Ru, Ir and Rh, and metal oxide catalysts, such as RuO_2_ and IrO_2_, have been considered as excellent catalysts for OER reactions, whereas for HER, Pt-based catalysts have been considered excellent [10]. However, the high cost and scarcity of noble metals hinder their commercial applications, in addition to their low stability and aggregation during electrochemical working conditions [11]. In this regard, various low-cost, transition-metal-based catalysts have been identified and explored for both OER and HER reactions. In particular, various 2D materials, such as graphene, layered double hydroxides (LDHs), graphitic carbon nitride (g-C_3_N_4_), transitional metal dichalcogenides (TMDs), have been explored [12,13,14,15,16,17].

## 2. Mechanism of Hydrogen Evolution Reaction

**Figure 2:**
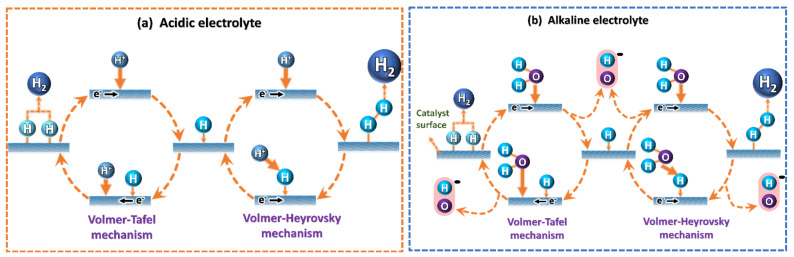
Schematic illustration of the hydrogen evolution reaction mechanism in (**a**) acidic and (**b**) alkaline media. HER.

The traditional HER mechanism utilizes the Volmer–Heyrovsky or Volmer–Tafel pathways, characterized by their Tafel slopes [18] (Figure 2 and Table 1).

In the acidic medium, HER starts with the adsorption of atomic hydrogen onto the electrode surface by the reduction in H^+^ with the help of an electron. In the second step, another H^+^ is reduced with the help of an electron, together combining the previously adsorbed atomic H to form H_2_. Alternatively, H_2_ may also form by combining two adsorbed atomic H, directly [19]. In total, HER is a two-electron transfer reaction, as shown below. In the alkaline medium, HER begins with water reduction to H* and OH^−^. In the second step, another water molecule dissociates to H* and OH^−^; simultaneously, two atomic H* combine to form H_2_.

**Figure 3:**
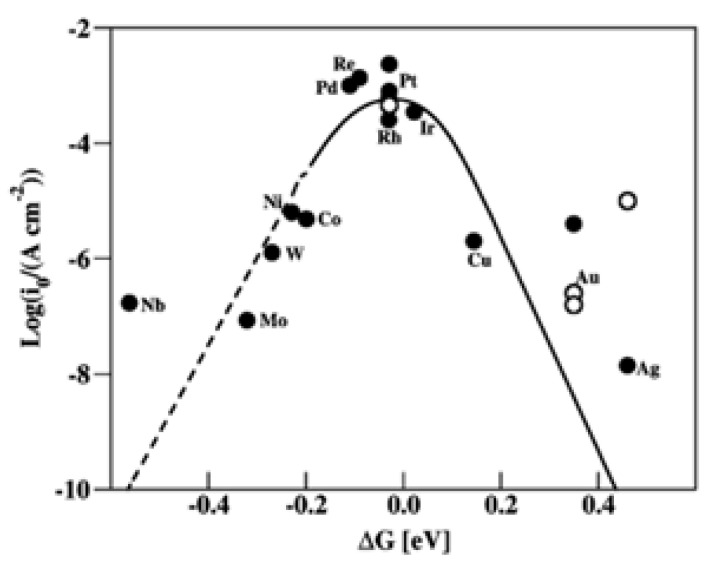
HER Volcano plot for metals (Reprinted with permission from Ref. [15]. Copyright 2010, American Chemical Society).

As per the chemical reactions stated above, the adsorption of hydrogen (H*_ads_) is an important step, and the Gibbs free energy (ΔG_H_*) of hydrogen adsorption is the criterion/rate determining step for the initiation of the reaction. The ΔG_H_* varies depending on the type of metallic electrocatalyst. An ideal electrocatalyst is one with the best compromise between the adsorption and desorption energies. Too strong adsorption leads to Heyrovsky/Tafel as the rate determining reaction, whereas weaker adsorption leads to a Volmer reaction as the rate determining step. Figure 3 shows the Sabatier volcano plot (ΔG_H_* vs. their exchange current densities) for the adsorption and desorption of H on various metallic surfaces [20]. It can be seen that most of the Pt group/noble metals occupy the highest position in the volcano plots with the optimized ΔG_H_* and high exchange current densities. This indicates that all the noble metal catalysts and Pt perform excellent HER kinetics, with nearly zero ΔG_H_* values. While the metallic catalysts on the left of the Pt group metals adsorb too strongly ΔG_H_* < 0, the metallic catalysts on the right of the volcano plots adsorb too weakly ΔG_H_* > 0, affecting the desorption and adsorption of the H atoms, thus ultimately affecting the overall HER kinetics.

## 3. Effect of Support Materials on the HER

In addition to the type of metal as an active site (as shown in Figure 3), the kinetics of HER is also influenced by the support material on which the metallic nanoparticles are dispersed. Support materials plays a key role in improving HER catalytic activity in a number of ways. The support material helps in the even distribution on the nanoparticles, thereby mitigating nanoparticle aggregation and enhancing the electrochemical active surface area of the nanoparticles [21]. Catalyst support acts as a mediator for the electron transport pathway into/out of the catalyst. For any material to be considered as catalyst support, it should possess some prerequisites, such as (i) a suitable surface area for the dispersion of nanoparticles, (ii) high electronic conductivity to minimize the ohmic losses, (iii) electrochemical stability (corrosion resistance), (iv) good metal-support adhesion, (v) suitable porosity. Therefore, the type of catalyst support highly influences the kinetics and stability of the catalyst. In general, carbon materials, such as carbon black, carbon nanofibers, graphene, carbon nanotubes, heteroatom (N, B, P, S, F, etc.) doped carbons, are used as support materials owing to their balanced electronic conductivity, surface area and porosity [22,23,24,25,26]. Nonetheless, carbon material materials are subjected to severe degradation (carbon corrosion) in the acidic and alkaline electrolytes [27]. Carbon corrosion leads to a loss of electronic contact, detachment of the supported nanoparticles and aggregation of nanoparticles, thereby reducing the kinetics of HER over longer periods of operation [28]. In this regard, various non-carbon supports have been explored as excellent and corrosion-resistant catalyst supports, such as transition metal oxides/carbides/nitrates. In comparison with traditional carbon-based supports, strong metal-support interactions between non-carbon supports and metallic nanoparticles have been found and well established by different researchers [29,30].

Novel 2D layered transition metal carbides/carbonitrides/nitrides, popularly known as MXenes, discovered by Drexel University, Philadelphia, United States researchers in 2011, have been gaining remarkable interest as support material for various electrochemical reactions, such as oxygen reduction reaction [31], methanol/ethanol oxidation reaction [32], nitrogen reduction reaction [33], sensors [34], carbon dioxide reduction reaction, supercapacitors [35], OER and HER [36], photocatalytic degradation of organic pollutants [37], improved mechanical and tribological properties [38], photooxidative performance for heavy metal removal [39] and hydrodeoxygenation reactions [40]. In this review article, we emphasized the importance of MXenes as catalyst/support material for HER catalysis. We briefly discussed the structure of MXenes, surface chemistry, stability and HER catalytic properties of MXenes-based electrocatalysts, followed by a summary of recent advances in MXenes electrocatalysts and HER kinetics, both from the theoretical and experimental point of view. Finally, we also proposed future research perspectives and directions for catalytic activity improvements.

## 4. MXene—Structure

MXenes are synthesized from their parent bulk material M_n+1_AX_n_, where M = transition metal, A is an A-group element, and X is C and/or N, n = 1, 2, 3. These M_n+1_AX_n_ materials are then simplified with the term MAX. The bulk MAX materials are structurally hexagonal, where the layers of the edge-shared M6X-octahedra are interleaved with layers of “A” elements, which are located at the center of the trigonal prisms. The “n” value indicates the number of “M” layers separating the A layers. The MAX phases possess unique properties, such as strong, mixed metallic-covalent M-X bonds on one side and weaker M-A bonds on the other sides. This unique structure enables breaking the weaker M-A bonds and maintaining the strong M-X bonds, selectively. Apparently, the MXenes are synthesized by treating the bulk parent material MAX with strong acids, such as NH_4_HF_2_, LiF + HCl and HF, which selectively etches the A layer to produce MXenes (M_n+1_X_n_T_n_). MXenes are a type of layered materials composed of transition metal (TM) carbide/nitride/carbonitride, represented by an empirical formula M_n+1_X_n_T_n_, (M = TM, X = carbon or nitrogen, T = -OH, -O and -F and n = integers 1, 2, 3) [41,42,43]. MXenes have three representative structures, namely, M_2_XT_x_, M_3_X_2_T_x_ and M_4_X_3_T_x_, with *n* layers of X elements covered by n + 1 layers of M [44]. There have been a number of review articles published in recent times describing the structure and properties of MAX phases and M_n+1_X_n_T_n_; they can be found in References [45,46,47] for detailed information.

## 5. Surface Chemistry and Stability of MXenes

The surface of MXenes offers a wide variety of functionalities (-F, -OH, -O, etc.) that are originated from the synthesis process, especially during the HF etching of the MAX phase. Various DFT calculations suggest that MXenes show a significant gain in negative formation energies after HF treatment, which indicates the formation of strong bonds between the surface of the M layer and chemical functionalities [48] by the following reactions.

Ti_3_AlC_2_ + 3HF → AlF_3_ + 3/2 H_2_ + Ti_3_C_2_

Ti_3_C_2_ + 2H_2_O → Ti_3_C_2_(OH)_2_ + H_2_

Ti_3_C_2_ + 2HF → Ti_3_C_2_F_2_ + H_2_

Recent studies revealed that some of the -OH terminations eventually undergo dehydrogenation reaction to form -O termination by the following reaction [49].

Ti_3_C_2_(OH)_2_ → Ti_3_C_2_O + H_2_O

There are many ways these surface functionalities affect the properties of MXenes, such as hydrophilicity/hydrophobicity, stability and electronic conductivity [50,51,52]. The thermodynamic stability of MXenes highly depends on the type of surface functional groups and follows the order of Ti_3_C_2_O_2_ > Ti_3_C_2_F_2_ > Ti_3_CH_2_O > Ti_3_C_2_H_2_ [53]. Further, long storage of MXenes also allows the structural phase transformations. A gradual oxidation of MXenes starts at the F-terminations into oxy-fluorides, leading to increased -O terminations along with the decreased -F terminations [54]. MXenes are also very sensitive toward oxygen, H_2_O and temperature. In the presence of O_2_, MXenes undergo structural phase transformations. For example, the colloidal Ti_2_C_2_T_x_ stored in an open atmosphere gradually transforms into TiO_2_, even at room temperature. Further, the smaller the flake size of Ti_2_C_2_T_x_, the faster the transformation. Therefore, it is always recommended that MXenes are stored in a colloidal form in dark and under low temperatures or as powdered MXenes in the inert atmosphere to ensure their intact structural stability [55]. Higher temperature is also known to have a definite effect on the structural phase of MXenes. A temperature of 200 °C and below is considered a safe temperature, without any disturbance to the layered Ti_2_C_2_T_x_ MXenes structure. Higher temperatures and oxygen atmosphere lead to the transformation of Ti_2_C_2_T_x_ to the anatase TiO_2_. In contrast, heat treatment in reducing atmospheres, such as H_2_/Ar, H_2_/N_2_, H_2_, is found to induce very minimal structural change in MXenes [56].

The electronic conductivity of MXenes is highly dependent on the density of the surface functional groups; the higher the density, the lower the electronic conductivity. Because the introduction of surface functional groups is inevitable with etchants such as HF, a careful optimization of the synthesis conditions is very important for applications where the electronic conductivity of the materials matters. For this purpose, there have also been studies to tailor the electronic properties of MXenes by tailoring the terminations and intercalation of the other ions into MXenes structures. One of the best approaches is to post treat the MXenes after the etching process. For example, the post treatment of MXenes by KOH or CH_3_CO_2_K, is found to reduce the -F terminations [57]. The other way is to perform a post heat treatment of MXenes in H_2_ atmosphere, which greatly reduces the -O or -OH terminations in the MXenes by the following equation [58].

Ti_3_C_2_(O)_2_ + 2H_2_ → Ti_3_C_2_ + 2H_2_O

Recently, vacuum annealing of MXenes was found to be one of the best ways to defunctionalize the surface groups and restore the electronic conductivity of Mxenes [59]. Therefore, choosing the right etchant, heat treatment temperatures, drying and storage of MXenes are very important to maintain their intact structure and stability for relevant applications.

## 6. MXene-Based Electrocatalysts for the HER

In general, high-cost precious metals, such as Pt and R/C, are used for HER due to their excellent kinetics and stability. However, high prices and low abundance are the major obstacles for the commercial use of precious metals. Therefore, research has been devoted to developing cheap electrocatalysts composed of non-precious metals/carbons, such as transition metal phosphides, nitrides, sulfides, oxides, and even electrocatalysts with no metal—so-called metal-free catalysts—have been developed [60,61,62,63,64]. However, there are still problems with non-precious-metal catalysts, such as poor electronic conductivity and low stability. In this regard, MXenes could hold potential properties, such as (a) their excellent metallic conductivity (up to 10,000 S cm^−1^), which facilitates faster charge-carrier transfer during HER that guarantees the high electronic density around the active sites; (b) structural integrity due to a layered structure; (c) corrosive resistance properties due to Ti, Mo, V in combination with C and N, which guarantee long-term stability in aqueous electrolytes; (d) intrinsic hydrophilicity, which ensures adequate contact with water molecules; (d) a large surface area to host the guest metal nanoparticles and expose maximum surface area for a reaction [27].

In addition, MXenes offer great stability over traditional Pt/C catalysts. In general, Pt/C catalysts are prone to undergo corrosion under acidic and alkaline conditions, which leads to Pt nanoparticle agglomeration. The traditional carbon, i.e., the Vulcan carbon, used as support for Pt nanoparticles is known to be extremely sensitive to carbon corrosion due to its poor crystallinity [65]. Further, it is well established that the interaction between the metal and carbon support is weaker in Pt/C [66]. In this regard, MXenes, as catalysts or catalyst supports, could offer potential stability due to the high crystallinity of MXenes and adequate bonding between C and M, and a lower density of unstable surface functionalities compared to Vulcan carbons. For example, Ti-based MXenes Ti_3_C_2_T_X_ bond strongly between Ti and C, mitigating the corrosion of carbon. The Ti in the catalyst provides ample durability under oxidative and acidic environments. Further, the strong bonding between Ti and C generates unique electronic properties at the Ti-C heterojunctions. In addition, the -F termination has been proven to be highly stable under a wide range of operating potentials; therefore, it could prevent the corrosion and aggregation of metallic nanoparticles, thus offering long-term stability compared to the traditional Pt/non-Pt carbon catalysts. Considering the above-mentioned advantages, various MXenes-based electrocatalysts have been synthesized using various strategies, such as modifying the surface terminal groups, metal doping, metal nanoparticles deposition and hybridization of MXenes with other carbon and non-carbon 2D materials. This review summarizes the recent developments of MXene electrocatalysts for HER, both from the theoretical and experimental research point of view.

### 6.1. Theoretical Perspectives on Kinetics and HER Mechanism of MXenes

Ab initio theoretical analysis has been gaining special attention in recent years, especially the density functional theory (DFT) calculations. For energy conversion reactions, DFT calculations help in identifying and predicting the active electrocatalysts together with the possible reaction mechanism and pathway based on the Gibbs free energy diagram, formation energy and volcano plots [67]. This section highlights the theoretical studies on MXene electrocatalysts. Bai et al. [68] investigated the HER activity of 20 different MXenes of two different families of MXenes, namely M_2_NO_2_, M_2_CO_2_ (M = Sc, Ti, V, Cr, Zr, Nb, Mo, Hf, Ta and W), using the Fermi abundance model. An ideal catalyst should have zero (ΔG_H_). When the H coverage analysis (25%) reveals M_2_NO_2_ (M = M = Ti, V, Cr, Nb and Ta) as the catalyst, the ΔG_H_ value is between −0.25 and 0.25 eV. In particular, Ti_2_NO_2_ and Nb_2_NO_2_ show close ΔG_H_ values with those of Pt (1 1 1), i.e., 0 eV, suggesting that Ti_2_NO_2_ and Nb_2_NO_2_ MXenes exhibit close HER activity to that of Pt, whereas M_2_NO_2_ (M = Sc, Zr, Mo, Hf and W) show high ΔG_H_ values, suggesting their low intrinsic HER activity. In the case of the carbide type of M_2_CO_2_ MXenes, the Ti_2_CO_2_ and W_2_CO_2_ catalysts show optimal ΔG_H_ (~0.12 eV). Similar observations are also drawn for the exchange current densities vs. ΔG_H_ volcano plots. Among all the catalysts, the Ti_2_NO_2_ and Nb_2_NO_2_ catalysts occupy the top of the curve, exhibiting larger exchange current densities (10^−17^ A/site) than the Pt (1 1 1) catalyst (10^−18^ A/site). In terms of the HER mechanism, Ti_2_NO_2_ and Nb_2_NO_2_, the Volmer–Heyrovsky mechanism is found to be preferred.

**Figure 4:**
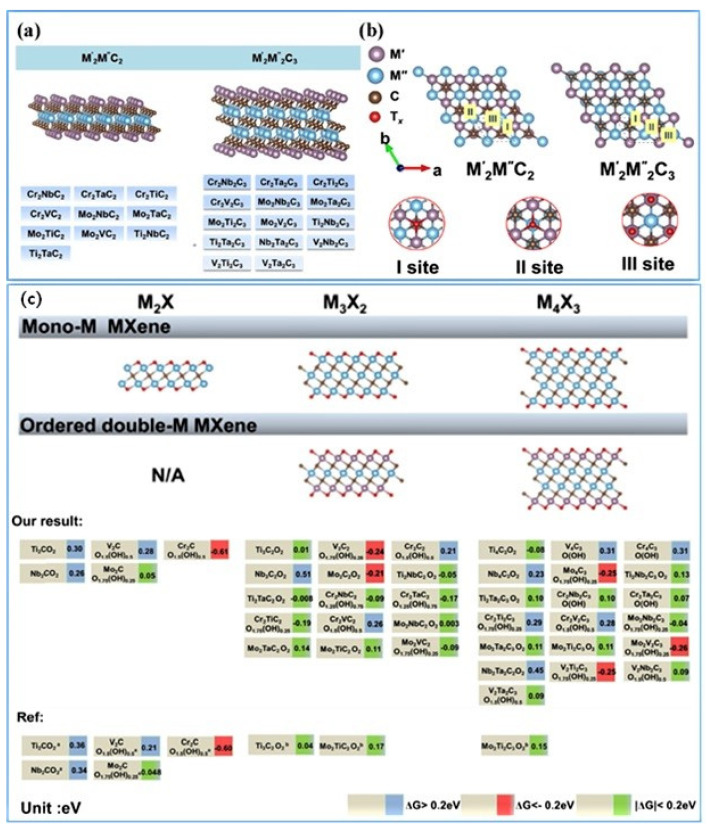
(**a**) List of ordered double TM carbides investigated in this work. (**b**) Atomic structure of M2′M″C2 and M2′M2″C3 viewed from the top. The “I site”, “II site” and “III site” represent three high-symmetry adsorption sites for -O and -OH termination on the outermost layer. Color code: M′ (blue), M″ (purple), C (brown) and Tx (red). (**c**) Calculated Gibbs free energy (in eV) for single M2′C, M3′C2 and M4′C3 (M′ = Ti, V, Cr, Nb and Mo) carbide MXenes and corresponding values for investigated double TM MXenes. (Reprinted with permission from Ref. [70]. Copyright 2020, American Chemical Society).

Huang et al. [69] investigated the transition metal carbonitrides M_3_CN type of MXene for ΔG_H_, and the electronic charge transfer on the surface terminated and un-terminated for HER kinetics. A detailed analysis reveals that Ti- and Nb-based MXenes (Ti_3_CNO_2_ and Nb_3_CNO_2_) show excellent HER kinetics. The calculated ΔG_H_ values are found to be negative (−1.173 to −0.709 eV) for all the investigated M_3_CN types. Further, the H adsorption is stronger on the N side of the MXene than on the C side. Further, the O-terminations are found to be beneficial for enhancing the HER, as can be seen from the smaller ΔG_H_ < 0.2 eV for Nb_3_CNO_2_ and Ti_3_CNO_2_, especially with Nb_3_CNO_2_. Further, the volcano plots suggest that Nb_3_CNO_2_ positions close to Pt. Jin et al. [70] investigated 2D ordered double transition-metal carbides (MXenes) with the chemical composition of M_2_′M″C_2_T_x_ and M_2_′M_2_″C_3_T_x_; M′ and M″ with two different metals (M′ = Cr, V, Ti, or Nb; M″ = Nb, Ta, Ti or V; and T = O and/or OH) by a detailed DFT analysis. A total of 18 different carbide species of different combinations were investigated, of which a double transition-metal carbide Mo_2_NbC_2_O_2_ showed the lowest overpotential. The volcano plots revealed that the HER performance of the double TM carbide MXenes strongly depends on the type of surface metal, and based on the performance, the investigated catalysts were grouped under three different categories, i.e., (i) O-terminated Mo-, Ti-based Mxenes with moderate adsorption for hydrogen species, corresponding with higher HER activity; (ii) O-terminated V- and Cr-based MXenes with strong adsorption for hydrogen species, corresponding with lower HER activity; (iii) MXenes with Ta and Nb with too weak hydrogen adsorption energies, corresponding with their unsuitability as HER catalysts. Further, in the case of double layer transition-metal carbide MXenes, it is observed that the type of metal in the inner layer has no significant effect on the HER activity. Further, it is found that -O, -OH and -F terminations help more in hydrogen adsorption than the bare double TM MXenes. Figure 4 shows the optimal 18 types of MXenes with the ideal overpotential of less than 0.2 V, derived from the DFT analysis.

**Figure 5:**
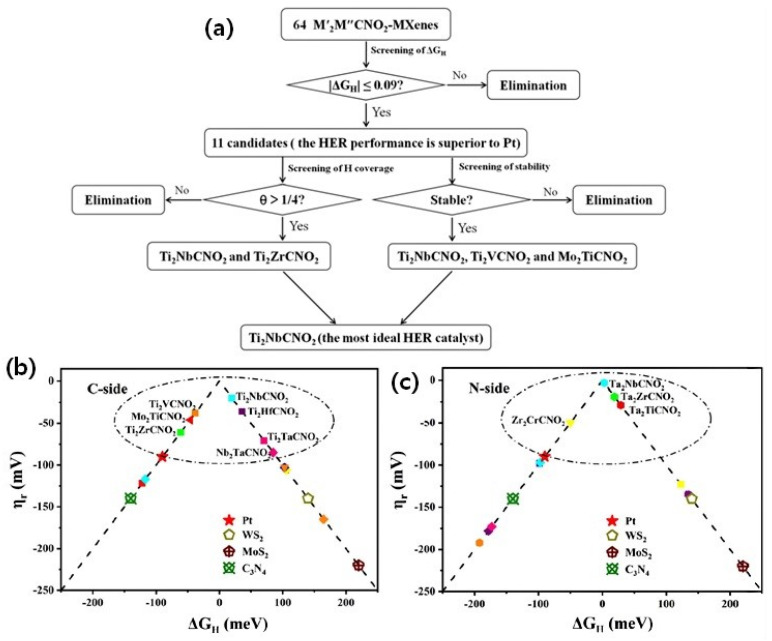
(**a**) Screening workflow. The screening process of HER catalysts from 64 M′_2_M″CNO_2_-MXenes. The volcano plotted for all 64 M′2M″CNO_2_-MXenes of (**b**) C side (**c**) N side. The different shapes represent different M′, and different colors represent different M″ (red, orange, yellow, green, cyan, blue, purple and pink represent Ti, V, Cr, Zr, Nb, Mo, Hf and Ta, respectively). Adapted from Ref. [71], (Open Access).

In another DFT study by Zeng et al. [71], they investigated 64 different types of double transition metal carbonitrides M′_2_M″CNO_2_ (M′ = Ti, V, Cr, Zr, Nb, Mo, Hf, Ta; M″ = Ti, V, Cr, Zr, Nb, Mo, Hf, Ta). Among the 64 types, 11 types of M′_2_M″CNO_2_ catalysts were found highly active in terms of Pt; regarding stability, Ti_2_NbCNO_2_, Mo_2_TiCNO_2_ and Ti_2_VCNO_2_ were found to be highly stable. The screenings of the most stable catalysts for HER are based on the Gibbs free energy calculations, as per the flow chart given in Figure 5 volcano plots. Similar to the earlier study [70], the type of metal on the outer layer was more important for enhancing the HER activity than the inner metals; especially when the outer layer was Ti or Nb, the HER activity was found to be higher than the other metals. Furthermore, the MXenes M′_2_M″CNO_2_ with the C side are found to be optimum for H adsorption compared to the N side. Further, the volcano plots indicate that both on the C side and the N side, together, the 11 types of M′_2_M″CNO_2_ catalysts show the best HER activity, with the best pick of the M′_2_M″CNO_2_ catalyst with ΔG_H_ values close to 0.003 eV, which is the best catalyst with almost zero ΔG_H_, showing its potential as an extraordinary HER catalyst. It is important to understand the formation energy and stability of the investigated MXenes due to the fact that the theoretically screened catalysts need to be able to synthesize in the laboratory conditions. The stability or formation energy analysis (E*_formation_*) reveals that of the 11 best screened catalysts, Ti_2_NbCNO_2_, Ti_2_VCNO_2_ and Mo_2_TiCNO_2_ catalysts showed negative formation energies, indicating that these types of M′_2_M″CNO_2_ are thermodynamically stable and can be synthesized experimentally. In addition to the traditional DFT studies, recently, machine-learning and high-throughput studies have been employed to screen and identify various possible MXenes for HER [72,73]. In summary, the DFT analysis of the single/double layered transition metal carbides/nitride reveals that there are tremendous opportunities to explore the different types and combinations of MXenes experimentally based on the DFT clues.

### 6.2. Experimental Perspectives on Kinetics and HER Mechanism on MXenes

#### 6.2.1. Pristine MXenes for HER

**Figure 6:**
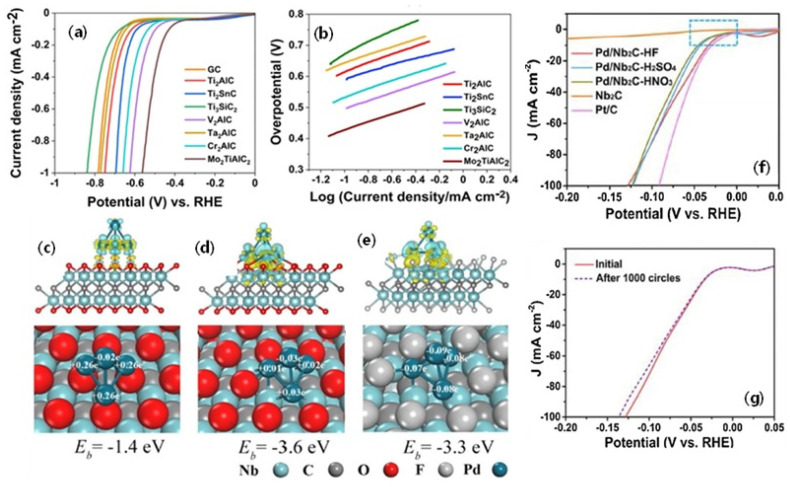
(**a**) Linear sweep voltammograms of GC, MAX phases at a scan rate of 5 mV s^−1^. (**b**) Tafel plots of MAX phases (Adapted from Ref. [74], Open Access). Charge density differences, binding energies, and Bader charges of Pd_4_ cluster supported with O-terminated Nb_2_C (**c**), O-terminated Nb_2_C with one O vacancy (**d**), F-terminated Nb_2_C with one F vacancy (**e**), cyan (yellow) isosurfaces indicate depletion (addition) of 0.005e/Å^−3^. The cyan, gray, red, silver, deep-blue spheres represent the Nb, C, O, F and Pd, respectively. (For interpretation of the references to color in this figure legend, the reader is referred to the web version of this article). Electrochemical performance of the supported nano catalysts (**f**) HER polarization curves (**g**) Polarization curves Pd/Nb_2_C-HF before and after 1000 cycles testing (Reprinted with permission from Ref. [76] Copyright, Elsevier).

Kumar et al. [74] investigated the HER activity of the pristine MAX phase materials of seven different types, namely, Ti_2_AlC, Ta_2_AlC, Ti_2_SnC, Ti_3_SiC_2_, V_2_AlC, Mo_2_TiAlC_2_ and Cr_2_AlC in 0.5 M H_2_SO_4_. The MAX phases of the respective compounds are used as such without etching of the M layer to understand the intrinsic HER activity of MXenes (Figure 6a,b). Half-cell studies indicate that among the seven types of MXenes, double layer MXene Mo_2_TiAlC_2_ showed enhanced HER activity followed by V_2_AlC. The overall order is as follows: Mo_2_TiAlC_2_ > V_2_AlC > Cr_2_AlC > Ti_2_SnC > Ti_2_AlC > Ta_2_AlC > Ti_3_SiC_2_. This order clearly indicates that the MXenes HER activity is based on the type of surface metal atoms. Further, the double metal atom layer MXenes seem to outperform single atom layer MXenes, as evidenced by the higher HER activity of Mo_2_TiAlC_2_ than the V_2_AlC and traditional Ti_2_AlC. In the Mo_2_TiAlC_2_, the sandwiched Ti atoms between the two layers of Mo, which are, in turn, adjacent to the detachable Al, generate the potential stacking structure made up of Mo-Ti-Mo-Al-Mo-Ti-Mo. The exposure of the Mo atoms sandwiched by Ti generates unique surface and electronic properties compared to traditional Ti_2_AlC. This unique structural arrangement is found to be the responsible factor for the enhanced HER activity of the Mo_2_TiAlC_2_ compared to the other types of MXenes. The Mo_2_TiAlC_2_ showed an overpotential of −0.57 V vs. RHE, which was higher among all the other types of MXens. This clearly indicates that MXenes composed of Mo, Ti, V are one of the best MXenes catalysts for HER activity.

#### 6.2.2. Effect of Surface Functionalization of MXenes for HER

It is well known that the etching of the parent MAX phase inevitably introduces various functional groups to MXenes, which includes -F, -O and -OH. These surface functional groups are known to enhance the stability and metal support interaction for several electrochemical reactions, such as ORR, OER and HER. DFT studies described in the above sections also evidenced that the surface terminated/functionalized MXenes’ lower HER overpotential than the bare MXenes. Li et al. [75] investigated the effect of F-terminated nanosheets of MXenes on HER and found a direct relationship with the content of -F. In a comparison study, the Ti_2_CT_x_ with less -F termination and the etched Ti_2_CT_x_ are reacted with KOH to remove the -F terminations and to remove the -O/-OH functional groups. The Ti_2_CT_x_ is further annealed at high temperatures (200 °C for 2 h). The HER analysis of the three catalysts shows that the Ti_2_CT_x_ terminated with -F shows better ORR activity, indicating that the presence of -F terminations is quite significant for HER activity. The Ti_2_CT_x_ devoid of the -O/-OH functional groups did not show significant change in the overpotential, whereas alkalized Ti_2_CT_x_ showed a drastically reduced overpotential, clearly indicating the significance of -F terminations. F-surface termination not only affects pristine MXenes’ HER activity; it also affects the supported metal nanoparticles. Zhang et al. [76] investigated the effect of -O/-OH and -F terminated Pd-supported Nb_2_CT_x_ on HER activity. From the DFT studies, it was revealed that Pd_4_ clusters absorbed on Nb_2_CF_2_-F_v_ and Nb_2_CO_2_-O_v_ supported the binding energy of −3.3 eV and −3.6 eV. On the contrary, Bader charge analysis revealed that there were 0.32 and 0.03 e^−^ transferred between Pd_4_ and Nb_2_CF_2_-F_v_ and Nb_2_CO_2_-O_v_, respectively (Figure 6c–e). Experimental HER analysis also revealed that the HER activity of the Pd_4_-supported Nb_2_CF_2_-F_v_ was strongly dependent on the surface functional groups. The obtained HER η of Pd/Nb_2_C-HF, Pd/Nb_2_C-H_2_SO_4_ and Pd/Nb_2_CHNO_3_ were 34, 43 and 46 mV. The HER performance of the catalysts decreased either with increased -O content or decreased -F contents, clearly suggesting that the HER kinetics are dependent on the content and type of surface functional groups. The optimized Pd/Nb_2_C-HF catalyst also showed excellent stability, both in chronoamperometric and potential cycling analyses (Figure 6f,g).

#### 6.2.3. Heteroatom Doped MXenes for HER

**Figure 7:**
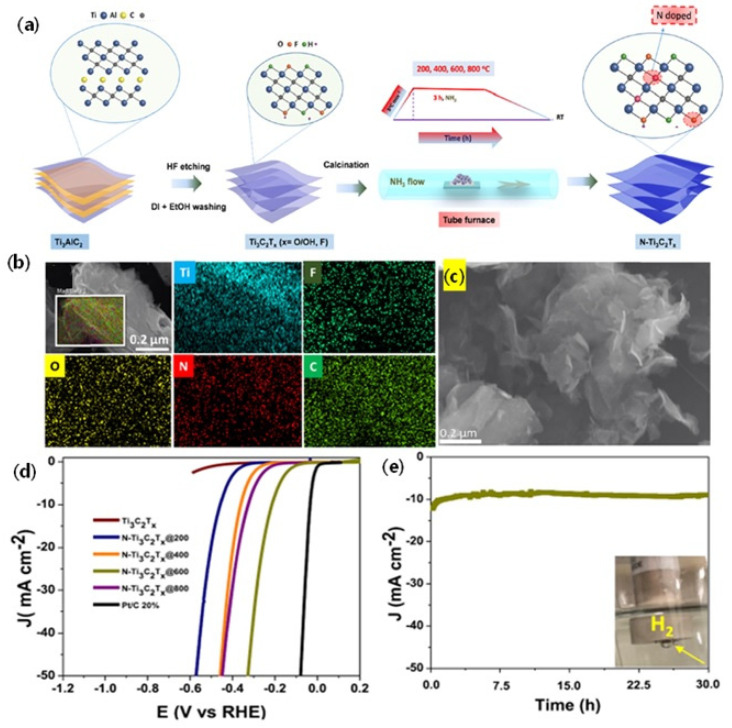
(**a**) Illustration of synthesis of N-doped MXene from the Ti_3_AlC_2_ MAX phase. (**b**) SEM–EDX elemental mapping of N-Ti_3_C_2_T_x_600 and (**c**) SEM image of delaminated N-Ti_3_C_2_T_x_@600. (**d**) HER polarization curves of Ti_3_C_2_T_x_ and a series of different N-doped Ti_3_C_2_T_x_ catalysts. (**e**) Test at a constant overpotential of −0.19 V (inset: photo of a rotating disk electrode (RDE) with the produced H_2_ bubbles on its surface). Reprinted with permission from Ref. [78]. Copyright 2019, American Chemical Society.

It is believed that doping of the heteroatoms, such as N, S and P, etc., with the MXenes enhances the chemical, electrochemical and electronic properties, which are beneficial for HER reactions. Doping of the heteroatoms not only creates defects but also exerts a synergistic interaction with the deposited metal nanoparticles for enhanced HER kinetics. Yoon et al. [77] successfully doped N into the Ti_2_CT_x_ by the process of nitridation by using sodium amide (NaNH_2_) as the source of N at the temperature of 500 °C. The XPS analysis clearly revealed the successful introduction of N by the appearance of Ti-N_x_ bonding states. It is observed that N doping with the Ti_2_CT_x_ changes the electronic properties, which results in increased HER activity of N-Ti_2_CT_x_ compared to pristine Ti_2_CT_x_. At the current density of 10 mA cm^−2^, the N-Ti_2_CT_x_ exhibits the η of −215 mV, whereas the pristine N-Ti_2_CT_x_ shows the η of −645 mV vs. NHE, which represents a more than three-fold enhancement over the pristine N-Ti_2_CT_x_. In another study, Le et al. [78] synthesized N-doped Ti_3_C_2_T_x_ by using NH_3_ heat treatment at 800 °C (Figure 7a). The SEM images show a sheet-like structure of N-doped Ti3C2Tx along with the successful doping of N ascertained from the SEM elemental mapping analysis (Figure 7b,c). It is found that N doping with Ti_3_C_2_T_x_ introduces different bonding possibilities, such as N-H, Ti-N and O-Ti-N. In addition to the Ti-N configurations, it is observed that N-H and O-Ti-N also change the electronic properties of Ti_3_C_2_T_x_, which leads to the optimized ΔHad* value close to zero (0) eV by DFT calculations, indicating the potential of N-doped Ti_3_C_2_T_x_ as excellent HER catalyst. In support of the DFT calculations, the N-doped Ti_3_C_2_T_x_@600 catalyst showed the η of 198 mV at 10 mA cm^−2^, several orders higher than the pristine Ti_3_C_2_T_x_ catalyst. The enhanced HER activity was associated with the synergistic effect on the electronic modification of the Ti_3_C_2_T_x_ due to N-doped active sites, such as N-H, Ti-N and O-Ti-N. Through a detailed XPS analysis, it was revealed that among the three possible N-doped active sites, N-H and O-Ti-N were found to be more important than Ti-N due to the fact that the % Ti-N in Ti_3_C_2_T_x_@800 was higher than Ti_3_C_2_T_x_@600; the former HER activity was less than the latter (Figure 7d,e). Further, higher calcination temperature was found to affect the % of N-H and O-Ti-N, as seen from the lower % of them in Ti_3_C_2_T_x_@800 than in Ti_3_C_2_T_x_@600. Therefore, it is reasonable to conclude that the optimized calcination temperature of 600 °C is essential for the optimized HER activity.

Han et al. [79] recently developed a simple ultrasonication strategy to precisely control the N doping with the Ti_3_C_2_T_x_ catalyst. The N doping is performed by ultrasonicating Ti_3_C_2_T_x_ in the presence of a mixture of ammonia and sodium borohydride solution at the temperature of 35 °C. With no further heat treatment, the N-doped Ti_3_C_2_T_x_ is obtained. It is believed that ultrasonic waves create tremendous defects for the adsorption of NH_3_ molecules. During ultrasonication, the removal of oxygen-containing functionalities provides the space for the doping of N atoms to create various N-containing functionalities, such as C-NH_2_, C-NH, Ti-N and Ti-NH_2_. Therefore, N doping occurs at the thermodynamically and kinetically unstable -O functional groups removed by the long ultrasonication process at 35 °C. SEM revealed that long-term sonication did not change the layered, structural stability of Ti_3_C_2_T_x_. When electrochemically evaluated for HER, the pristine Ti_3_C_2_T_x_ catalyst showed a large η of 575 mV, whereas N-Ti_3_C_2_T_x_-35 showed excellent enhancement, with a η of 163 mV. The Tafel slope analysis revealed a slope of 69 mV dec^−1^, indicating that the HER reaction mechanism in N-Ti_3_C_2_T_x_-35 is a type of Volmer–Heyrovsky reaction. Impedance spectroscopy also revealed the lowest resistance for N-Ti_3_C_2_T_x_-35 due to the doping of N, which helped in faster charge transfer during the electrochemical reactions. In addition to the enhanced HER activity, N-Ti_3_C_2_T_x_-35 also showed excellent stability, with almost zero degradation in the overpotential after 24 h, and a stable current with almost no loss in potential for 35 h. In summary, the doping of N with the Ti_3_C_2_T_x_-based MXenes is proved to be beneficial for enhanced HER kinetics and stability.

#### 6.2.4. Pt/Ti_3_C_2_T_x_-Based MXenes for HER

**Figure 8:**
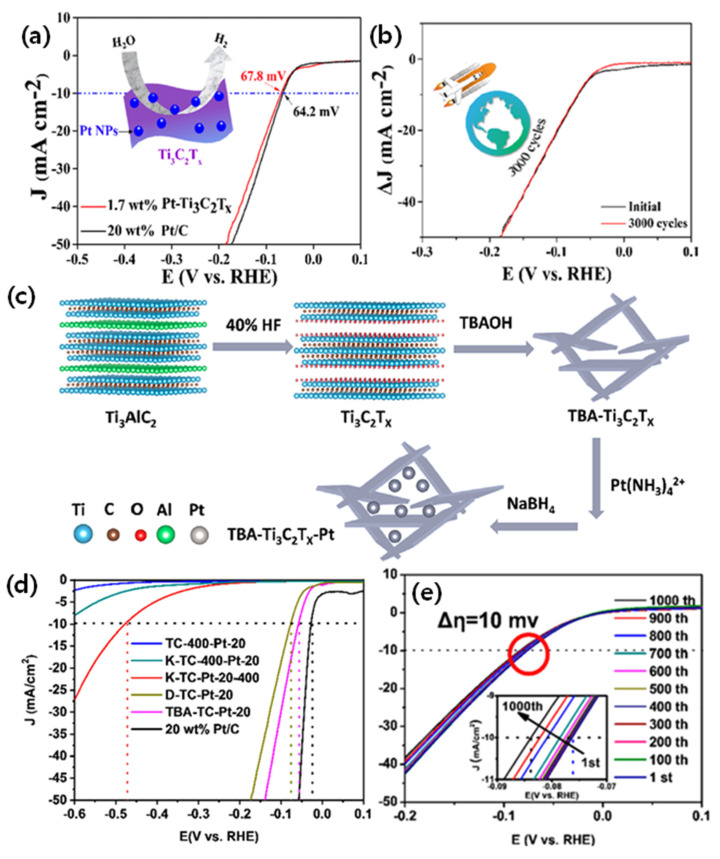
(**a**) Linear sweep voltammetry curves (**b**) Plotting of current—density difference (ΔJ) against the voltage scan rate of 1.7 wt% Pt-Ti_3_C_2_T_x_ initial and 3000th cycle. (Reprinted with permission from Ref. [54]. Copyright 2020, American Chemical Society). (**c**) Schematics of the preparation process of TBATi_3_C_2_T_x_-Pt. (**d**) Polarization curves of Pt deposited on the supports in 0.5 M H_2_SO_4_. (**e**) Polarization curves of TBA-Ti_3_C_2_T_x_-20 obtained for 1000 cycles (Reprinted with permission from Ref. [81]. Copyright 2019, American Chemical Society).

In general, Pt/C catalysts with Pt wt% ~20 are used in the current technology and as a comparative benchmark for assessing the other types of catalysts. However, 20 wt% of Pt is still high for practical application due to its high cost. Efforts have been undertaken to reduce the loading of Pt several-fold while at the same time maintain the high HER kinetics. Zhnag et al. [80] investigated the HER activity of low Pt loading supported on Ti_3_C_2_T_x_. The Pt deposition was carried out by the atomic layer deposition (ALD) technique. Pt NPs of 2 nm in diameter are deposited by controlled ALD cycles, and the obtained Pt/Ti_3_C_2_T_x_ of Pt wt% 1.7 catalyst showed almost equal HER activity to that of Pt/C 20 wt%. The TEM images clearly show that the Pt nanoparticles are homogeneously dispersed on the Ti_3_C_2_T_x_ support with a lattice fringe of 0.23 nm, corresponding to Pt (1 1 1). The 40Pt-TBA-Ti_3_C_2_T_x_ catalysts showed an overpotential of 67.8 mV@10 mA cm^−2^, whereas it was 64.2 mV for Pt/C catalysts, a mere 3.6 mV inferiority. This indicates that the low loading of Pt by ALD could deliver similar HER activity as that of a high loading of Pt/C. The high HER activity of 40Pt-TBA-Ti_3_C_2_T_x_ is further ratified by measuring the ECSA, which show a calculated value of 16.6 and 16.6 µF cm^−2^, which is almost close to the Pt/C catalyst (19.7 µF cm^−2^), despite the 18-times-lower Pt loading due to the fact the ALD gives homogeneous dispersion compared to the traditional wet impregnation of Pt, which results in agglomerations. The 1.7 wt% Pt-Ti_3_C_2_T_x_ catalyst showed excellent stability over 3000 cycles, with no visible change in the voltage (Figure 8a,b).

In another study by Yuan et al. [81], the Pt nanoparticles are supported on the Ti_3_C_2_T_x_ catalyst by a simple reduction in metallic precursors by a web impregnation method (NaBH_4_ reduction method), and the Pt-deposited Ti_3_C_2_T_x_ catalyst is then studied for HER reaction (Figure 8c). Various samples were synthesized, such as K-intercalated, tetrabutylammonium hydroxide (TBA) and the heat-treated (@400 °C) samples. Among all the samples, the TBA-TC-Pt-20 (corresponding to 1 wt% of the Pt) samples showed the best HER activity, whereas the heat-treated samples @ 400 °C showed the worst activity due to the partial damage to the Ti−C layers of the supports. XPS analysis revealed that the Pt4f peaks are shifted to higher binding energies, indicating the strong interaction between Pt and the Ti_3_C_2_T_x_ support, which in turn helps in the fast charge transfer burning the HER. The TBA-TC-Pt-20 catalyst showed a η of 55 mV, much higher than the other Pt catalysts. In addition, the TBA-TC-Pt-20 catalyst also showed admirable durability, with just 10 mV of the potential decrease after 1000 cycles (Figure 8d,e).

In a similar attempt to reduce the Pt loading, Wu et al. [82] developed a spray drying route to synthesize 3D crumbled Ti_3_C_2_T_x_ supported with the atomic Pt clusters as highly active HER catalysts. The spray drying technique could avoid the successful re-stacking of Ti_3_C_2_T_x_ and mitigate the agglomeration of the Ti_3_C_2_T_x_ nanosheets during the catalyst deposition process, thereby increasing the exposure of the electrochemical active surface area. The colloidal tetrabutylammonium hydroxide intercalated Ti_3_C_2_T_x_ nanosheets are mixed with the chloroplatinic acid solution, and the mixture is spray dried. Due to the presence of low-valance Ti^2+^/Ti^3+^, spontaneous reductions in the Pt^4+^ ions into Pt single atom/cluster are deposited on the Ti_3_C_2_T_x_ nanosheets. SEM and TEM images clearly showed the 3D crumpled Ti_3_C_2_T_x_ structure, on which the Pt clusters with homogeneous distribution were noticed. The Pt/ Ti_3_C_2_T_x_ MXene catalyst showed extraordinary HER activity, with nearly zero onset potential and a η of 34 mV @ 10 mA cm^−2^, nearly equal to Pt/C (37 mV). Further, the Pt loading of 2.9 wt% was achieved by the spray drying technique. When the HER activity is normalized by the mass, the Pt/MXene catalyst exhibits seven times over Pt/C. This is due to the high surface area of Ti_3_C_2_T_x_ and the subatomic/clusters of Pt, which can expose more Pt surface area for HER than typical Pt nanoparticles on carbon support. Such a high HER mass activity was also supported by the DFT calculations. The PDOS analysis revealed that the d-band center shifted nearer to the ideal fermi level, indicating that the Pt on TiCO-F offered optimized binding energy for hydrogen atoms, which was neither too strong/too weak for the chemical reaction. In addition, ΔG_H_* for Pt/TiCO-F was lower than the Pt (111), i.e., 0.05 eV vs. −0.18 eV. The shift in the d-band center and lower ΔG_H_* for Pt/TiCO-F demonstrated the high HER activity of it.

#### 6.2.5. Non-Pt/Ti_3_C_2_T_x_-Based MXenes for HER

**Figure 9:**
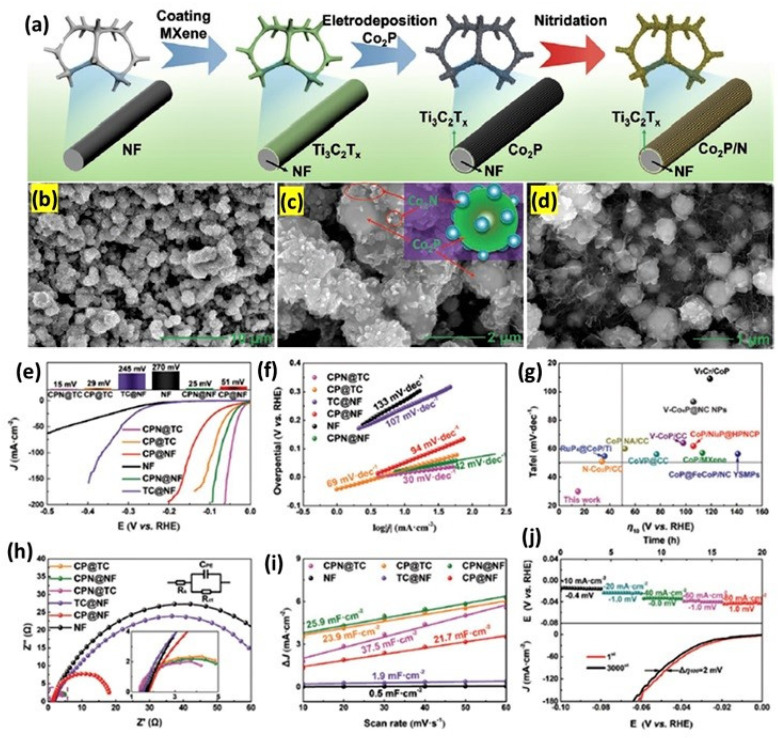
(**a**) Schematic illustration of the catalyst synthesis strategy. (**b**–**d**) SEM images of (**b**–**d**) CPN@TC. (**e**) Polarization curves and (**f**) corresponding Tafel slopes of CPN@TC, CP@TC, CP@NF, NF, CPN@NF and TC@NF. (**g**) Comparison of CPN@TC with reported electrocatalysts. (**h**) EIS patterns and (**i**) the C_dl_ of CPN@TC, CP@TC, CP@NF, NF, CPN@ NF and TC@NF. (**j**) Long-term chronopotentiometry response of CPN@TC at different current densities (−10, −20, −40, −60, −80 mA cm^−2^) and polarization curves for CPN@TC before and after 3000 cycles (Reprinted with permission from Ref. [84]. Copyright 2021, John Wiley and Sons).

Among the non-Pt-based catalysts, the transition metal-based sulfides, phosphides, nitrides and carbides have attracted attention recently due to a variety of electrochemical reactions, including HER. Especially when hybridizing the transition metal derivatives with the support materials, such as carbon and 2D materials, e.g., carbon nanotubes, graphene, etc., it is found that the support materials offer essential surface area and electronic conductivity, which together expose the catalytic active sites [83]. In this regard, a variety of transition metal derivatives are hybridized with MXenes and are explored as efficient electrocatalysts in efforts to reduce the Pt catalyst price. Lu et al. [84] synthesized a Co_2_P/N@Ti_3_C_2_T_x_ supported on nickel foam (CPN@TC) as an ultra-efficient HER catalyst. The Co_2_P/N@Ti_3_C_2_T_x_ with the industrial potential is synthesized by an electrodeposition method on Ni foam as the substrate. The SEM images show the Ni foam is covered by the nanosheets of Ti_3_C_2_T_x_. When the electrode potential is applied, the Co_2_P nanoparticles are grown in the Ti_3_C_2_T_x_ nanosheets (Co^2+^ + 2e^−^ → Co, 2Co + H_2_PO^2−^ + 2H^+^ + e^−^ → Co_2_P + 2H_2_O). Further, the Co_2_P nanostructures are then converted into nitrides by the process of nitridation by using ammonium carbonate decomposition. Nitridation is known to increase conductivity, and hence, it results in a better electrocatalytic activity. Nitridation also replaces some of the surface functional groups, such as -F, -O and -OH, together with the doping of N into the Co_2_P to form N-doped Co_2_P. Such multi-heterojunction interfaces in the Co_2_P/N@Ti_3_C_2_T_x_ could offer unique 3D interfacial electron transfer pathways for improved HER activity. The resulting Co_2_P/N@Ti_3_C_2_T_x_ showed extraordinary HER activity with a η of 15 mV @−10 mAcm^−2^ and a Tafel slope close to 30 mV dec^−1^. In terms of durability, the Co_2_P/N@Ti_3_C_2_T_x_ catalyst showed no obvious change in potential for 60 h of operation at −10 mAcm^−2^, and under potential cycling conditions, it showed a marginal loss of just 2 mV after 3000 cycles, indicating the potentiality of this catalyst in industrial applications (Figure 9). In another study, Han et al. [85] synthesized multi-dimensional hierarchical CoS_2_@MXene through a hydrothermal reaction followed by the sulfurization process. Nanowires of CoS_2_ were attached to Ti_3_C_2_T_x_ with no obvious agglomerations. XPS analysis showed the Co and S peaks shifted to a high binding energy when hybridized with Ti_3_C_2_T_x_, indicating the strong interaction between CoS_2_ and Ti_3_C_2_T_x_. However, the CoS_2_@MXene catalyst still showed a large overpotential of 175 mV@10 mAcm^−2^. Jiang et al. [86] synthesized transition metal-based chalcogenide core shell nanostructure Ti_3_CNCl_2_@CoS_2_ as an excellent HER catalyst. The Cl-terminated MXenes were synthesized by the molten salt method, and the CoS_2_ nanostructures were deposited by a hydrothermal synthesis process. Through a DFT analysis, the hybridization of CoS_2_ with Ti_3_CNCl_2_ effectively brought down the ΔG_H_* to −0.1 eV from their individual constituents of −0.89 and +1.86 eV, respectively. SEM images and elemental mapping indicated that the Ti, N, Cl were present at the center, whereas the Co and S elements were seen on the peripheral part of the core shell nanoparticles. With the optimized 7.63% of CoS_2_, the Ti_3_CNCl_2_@CoS_2_ catalyst showed improved HER activity compared to its constituent CoS_2_ with Ti_3_CNCl_2_.

Another transition metal, Ni, and its derivatives, such as NiP and NiSe_2_, have also attracted a lot of attention due to the fact that Ni derivatives are chemically stable and accelerate the HER kinetics [87]. Lu et al. [88] synthesized NiP/Ti_3_C_2_T_x_ supported by a 3D Ni foam by a hydrothermal method, followed by phosphorylation. The ultraviolet photoelectron spectroscopy (UPS) was used to understand the band structure and work function. It was observed that the valance band maximum (E_v_) of the NiP/Ti_3_C_2_T_x_ catalyst showed a larger value than Ti_3_C_2_T_x_. In addition, the work function of NiP/Ti_3_C_2_T_x_ was found to be lower (2.13 eV) than Ti_3_C_2_T_x_ (2.2 eV). Both the E_v_ and work function proved that the electrons in the NiP/Ti_3_C_2_T_x_ are more easily accessible for electrochemical reactions. The NiP/Ti_3_C_2_T_x_ catalyst showed a η of 135 mV @10 mA cm^−2^. Through a DFT analysis, the ΔG_H_* of NiP/Ti_3_C_2_T_x_ was found to be −0.28 eV, close to that of ΔG_H_* of Pt (−0.09 eV). In another study, Jiang et al. [89] synthesized octahedral NiSe_2_-supported Ti_3_C_2_T_x_ by a one-pot hydrothermal synthesis. The Ti_3_C_2_T_x_ nanosheets were mixed with the NiCl_2_-EDTA and the Se (KOH) solution, which forms the Ni-EDTA chelated complex, which, upon the hydrothermal conditions, forms the octahedra NiSe_2_ supported by MXenes. HRTEM images show the perfect single-crystal nature of NiSe_2_ with a d spacing of 0.27 nm corresponding to a (210) plane and 1.0 nm of Ti_3_C_2_T_x_ corresponding to a (002) plane. The strong coupling interaction between the Ti_3_C_2_T_x_ and NiSe_2_ is further established by the XPS analysis in which the Ni2p peaks shift to a higher binding energy by 0.3 and 0.6 eV Ni 2p_3/2_ and Ni 2p_1/2_, respectively. Nevertheless, NiSe_2_/Ti_3_C_2_T_x_ showed a large overpotential (200 mV @10 mA cm^−2^) when compared to all the other types of catalysts discussed in the earlier sections, indicating that selenides might have a lower HER activity over sulphides, phosphides and nitrides.

Guided by the DFT calculations, the doping of Nb with Ti_3_C_2_T_x_ modified the electronic conductivity of the support by lifting the fermi level, and, at the same time, alloying Ni and Co efficiently adjusted the M-H attraction, synergistically enhancing the HER activity (Du et al. [90]). The ΔG_H_* of the Ti_3_C_2_ and Nb-doped Ti_3_C_2_ catalysts is found to be −0.25 eV and −0.14 (O site nearby Nb-doped atoms) and −0.23 eV (O site nearby Nb-doped atoms), indicating that Nd-doped Ti_3_C_2_ could deliver enhanced HER activity. Further, by adding Co and Ni atoms, the ΔG_H_* values further decreased to more than half of the Nb-doped Ti_3_C_2_ catalyst. HR-TEM images show the multilayered, crumbled nanosheet morphology of Ti_2_._5_Nb_0.5_C_2_T_x_ and Ni_0.9_Co_0.1_ alloy nanoparticles of 5 nm are deposited. Electrocatalytic HER of the NiCo@NTM is evaluated in 1 M KOH. The IR corrected NiCo@NTM LSV curves show a η of 43.4 mV @ 10 mA cm^−2^, which is almost close to the benchmark Pt/C (34.4 mV). Further, when the current densities are normalized by the double layer capacitance (C_dl_) *J_cdl_ @* 1000 mA F^−1^, the NiCo@NTM catalyst exhibits a lower overpotential of 55.5 mV, higher than the Pt/C. Impedance spectroscopy also suggests a lower resistance to the NiCo@NTM catalyst. During the stability test, the NiCo@NTM catalyst also showed excellent durability over 50 h in the chronopotentiometry test. Therefore, it is reasonable that the NiCo@NTM catalyst is a promising non-precious-metal catalyst for HER in alkaline medium.

#### 6.2.6. Nb_2_CT_x_-Based MXenes for HER

**Figure 10:**
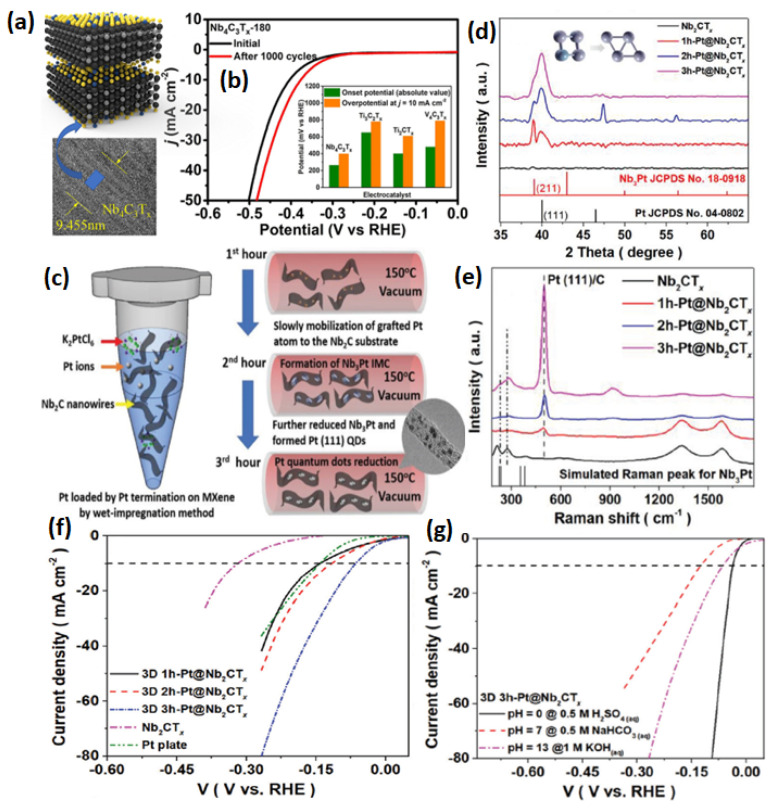
(**a**) Schematic of Nb4C3Tx. (**b**) Polarization curves of the Nb4C3Tx-180 electrode before and after 1000 cycles at 100 mV s^−1^ under an alkaline onset potential and overpotential at j @ 10 mA cm^−2^ in different MXenes conditions (Reprinted with permission from Ref. [91]. Copyright 2021, Elsevier) (**c**) Schematic for the fabrication of Pt QDs on Nb2CTx NWs. (**b**,**c**) Structural investigation of various Pt QDs on MXene by their (**d**) XRD patterns and (**e**) Raman spectra. (**f**) LSV for 3D Pt@Nb2CTx with different synthesis times (**g**) The LSV for 3D 3h-Pt@Nb_2_CTx in different electrolytes (Adapted from Ref. [92], Open access).

It is considered that the research on MXenes is in its infancy. Theoretical simulations suggest more than 70 types of MXenes are possible; however, until now, very few MXenes have been successfully synthesized and explored for electrochemical reactions. Among them, Ti_3_C_2_T_x_ has been the most explored MXene. However, recently, there have been other types of MXenes also gaining special attention. For example, Tan et al. [91] investigated the HER performance of pristine Nb_4_C_3_T_x_ in acidic and alkaline electrolytes. Similar to Ti_3_C_2_T_x_, the Nb_4_C_3_T_x_ MXene was synthesized from Nb_4_AlC_3_ by HF etching. The SEM images show that the compact Nb_4_AlC_3_ structure is loosened and exhibit the layered structure in Nb_4_C_3_T_x_, indicating the successful etching of the Al layer. The etching process was carried out at room temperature at different times in the HF solution, which was varied between 140 h to 220 h. An optimum time of 180 h was found suitable for sufficiently removing the Al layer, analyzed by calculating the ratio of I_MAX_/I_MXene_ (I_MAX_ and I_Mxene_ represents the diffraction peak intensity of (103) for Nb_4_AlC_3_ and (002) for Nb_4_C_3_T_x_, respectively). The Nb_4_C_3_T_x_-180 sample showed the lowest I_MAX_/I_Mxene_, which indicated that the Al layer was removed by stirring for 180 h. When comparing the HER activity of all the Nb_4_C_3_T_x_ treated at different time intervals, Nb_4_C_3_T_x-_180 showed better HER activity (398 mV @ 10 mA cm^−2^) than the other catalysts. However, the Nb_4_C_3_T_x_-180 HER activity was far behind the Pt/C catalyst. However, when the Pt nanoparticles were introduced to Nb_4_C_3_T_x_, the HER activity was enhanced by several-fold (Figure 10a,b).

Pang et al. [92] reported the nanowire morphology of 1D Nb_2_CT_x_ as a HER catalyst. The nanowire morphology of 1D Nb_2_CT_x_ was obtained by the electrochemical etching process. The 1D Nb_2_CT_x_ has a length of 100–400 nm and a width of 50 nm. The Pt is impregnated onto 1D Nb_2_CT_x_ by a process of wet impregnation, with Pt nanoparticle size of 2 nm. The resulting Pt@Nb_2_CT_x_ exhibited an overpotential of 33.3 mV in acidic and 65.1 mV in basic electrolytes @ 10 mA cm^−2^. The high HER activity was attributed to the strong metal support interaction between Pt and Nb_2_CT_x_, evidenced structurally, morphologically and through spectroscopic methods. The strong interaction also offers robustness and stability (Figure 10c–g). In another study, Fan et al. [93] mechanochemically synthesized Pt/Nb_2_CT_x_ and obtained excellent HER activity, close to the commercial Pt/C catalyst. Typically, the Nb_2_CT_x_ powder was mixed with the solution of PtCl_6_^2−^ and ball milled at the speed of 150 rpm for about 30 min. Then, the power was subjected to annealing at 600 °C for 2 h under the Ar gas. The TEM images show the nanoclusters of Pt were introduced on the Nb_2_CT_x_ of about 1.5 nm. During the annealing process, the Pt also alloyed with Nb to five Pt_3_Nb nanoparticles. The HER analysis shows that the Pt/Nb_2_CT_x_-600 catalyst showed excellent activity compared to the Pt/Nb_2_CT_x_ and Pt/C catalysts. At the current density of 10 mA cm^−2^, the Pt/Nb_2_CT_x_-600 catalyst exhibited a η of 5 mV, lower than the 6.2 mV for Pt/C. In addition, the Pt/Nb_2_CT_x_-600 catalyst also exhibited excellent durability, with no loss in η after 5000 cycles and a marginal loss in the relative current after 20 h. The excellent HER activity of the Pt/Nb_2_CT_x_-600 catalyst is attributed to the strong metal support interaction and homogeneous distribution of Pt and Pt_3_Nb nanoparticles on the Nb_2_CT_x_ support.

#### 6.2.7. V_4_C_3_T_x_-Based MXenes for HER

**Figure 11:**
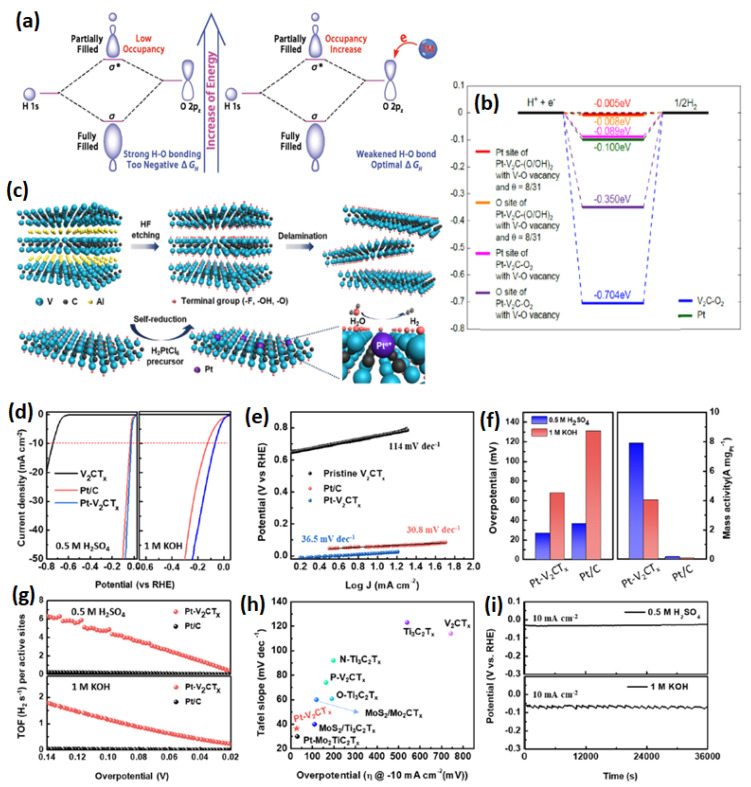
The scheme of modulating the HER performance of V_2_CO_2_ by introducing transition metal onto the surface. (**a**) The combination of H 1s orbital and O 2pz orbital forms a fully filled bonding orbital and a partially filled anti-bonding orbital, in which the occupancy of anti-bonding orbital will determine the strength of H O bond. (**b**) Structural characteristics of Pt-V_2_CT_x_ nanosheets (Adapted from Ref. [95] Open access). (**c**) Schematic illustration for synthesis of Pt-V_2_CT_x_ nanosheets. Electrocatalytic performance for Pt-V_2_CT_x_ and reference HER electrocatalysts. (**d**) HER polarization curves of pristine V_2_CT_x_, Pt/C (20%) and Pt-V_2_CT_x_, acquired using graphite rod as the counter electrode in 0.5 M H_2_SO_4_ solution (left) and in 1 M KOH solution (right). (**e**) Corresponding Tafel slope derived from a. (**f**) Comparison of overpotential (10 mA cm^− 2^) and mass activity for Pt/C (20%) and Pt-V_2_CT_x_ catalysts in 0.5 M H_2_SO_4_ (blue) and 1 M KOH (red). (**g**) Turnover frequency (TOF) of Pt/C (20%) and Pt-V_2_CT_x_ in 0.5 M H_2_SO_4_ solution (top) and in 1 M KOH solution (bottom). (**h**) Comparative graph for overpotential of related MXene-based HER electrocatalysts in acidic media. (**i**) Stability test of Pt-V_2_CT_x_ in 0.5 M H_2_SO_4_ solution (top) and 1 M KOH (bottom). (Reprinted with permission from Ref. [97]. Copyright 2021, Elsevier).

A new class of V_4_C_3_T_x_ MXenes has gained special interest in recent years due to its applications in lithium-ion batteries, as a positive electrode in sodium ion capacitors and as an electrocatalyst for CO_2_ capture [94]. However, for HER, it has been very rarely investigated. Ling et al. [95] predicted that highly catalytically active V_2_CO_2_ MXene supported Fe, Co and Ni for HER catalysis. As it is known that a synthesis of any type of MXenes needs to have obvious oxygen functionalities, therefore, the modeling was performed with oxygen terminations. When H adsorbs on V_2_CO_2_, the H 1s orbital and terminated O 2p_z_ orbital hybridize to form the bonding orbital and the antibonding orbital (σ and σ*). When the σ* occupancy is higher, the bonding strength of H will be higher. By introducing the metal atoms, which act as metal donors, a higher electron transfer occurs from metal atoms to -O, and thereby, there is a higher occupancy in the σ* that leads to O-H bonding. The higher charge on the O atom due to electron transfer from the metal center leads to decreased charge transfer from H to O; therefore, the interaction between O and H will be weakened. This promotes the optimal adsorption for HER kinetics (Figure 11a,b). When the transition metal (TM) supported V_2_CO_3_, the binding energies (*E*_b_) were observed to be higher than 1.0 eV, indicating a strong interaction between V_2_CO_3_ and TM. Among all the investigated TM, a significant promotional effect (0.7 e^−^ from TM to V_2_CO_2_) is observed for Fe, Co and Ni, and the calculated ΔG_H_ is from Fe to Co to Ni-V_2_CO_2_; therefore, it is concluded that the HER activity of Fe-V_2_CO_2_ is to be higher than the other TM-V_2_CO_2_ catalysts. Not many experimental studies are found to investigate the Vanadium-based MXenes. Tran et al. [96] investigated the HER activity of pristine V_4_C_3_T_x_. It is seen that the pristine V_4_C_3_T_x_ showed a η of 200 mV @ 10 mA cm^−2^. Park et al. [97] reported the atomic Pt deposited V_2_CT_x_. It is believed that HF etching creates the V vacancies, which act as metal immobilizing sites for Pt deposition (Figure 11c). A loading as low as ~0.88 wt% and the identification of Pt-C bonding are responsible for the excellent, Pt-like HER activity of the Pt/V_2_CT_x_ catalyst. The V vacancies that are naturally formed during the Al etching process are known to be highly unstable and reductive in nature. When PtCl_6_^2−^ ions are added to the exfoliated V_2_CT_x_, the PtCl_6_^2−^ are spontaneously adsorbed on the V vaccines and are reduced by the reductive nature of V vaccines to metallic Pt. The atomic distribution of the Pt atoms is revealed by the HAADF-STEM analysis in which the Pt atomic sites are seen with bright spots on the V_2_CT_x_ nanosheets. Further, it is seen that the Pt atoms are located at the V lattice plane, evidencing that V vacancies attract and stabilize the Pt atoms. The HER analysis reveals that the Pt-V_2_CT_x_ catalyst showed higher activity (27 mV of η @ 10 mA cm^−2^) than the Pt/C (36.5 mV of η @ 10 mA cm^−2^). While the Pt loading on V_2_CT_x_ is ~19 times (~0.88 wt%) lower than the commercial Pt/C (20 wt%), when normalizing the HER mass activity, extraordinarily, the Pt-V_2_CT_x_ catalyst was 50 times higher (7.88 A mg_pt_^−1^ @ η of −30 mV) than the commercial Pt/C catalyst (0.157 A mg_pt_^−1^ @ η of −30 mV). Similar statistics were also drawn, in turn, over the frequency analysis, in which the Pt-V_2_CT_x_ showed a TOF of 4.74 and 1.16 H_2_ S^−1^, whereas for Pt/C, it was 0.156 and 0.0226 H_2_ S^−1^ (Figure 11d–i). The HER activity obtained in this study is one of the best among several other types of MXenes, even higher than the P-doped V_2_CT_x_ [98].

#### 6.2.8. Mo_2_CT_x_-Based MXenes for HER

**Figure 12:**
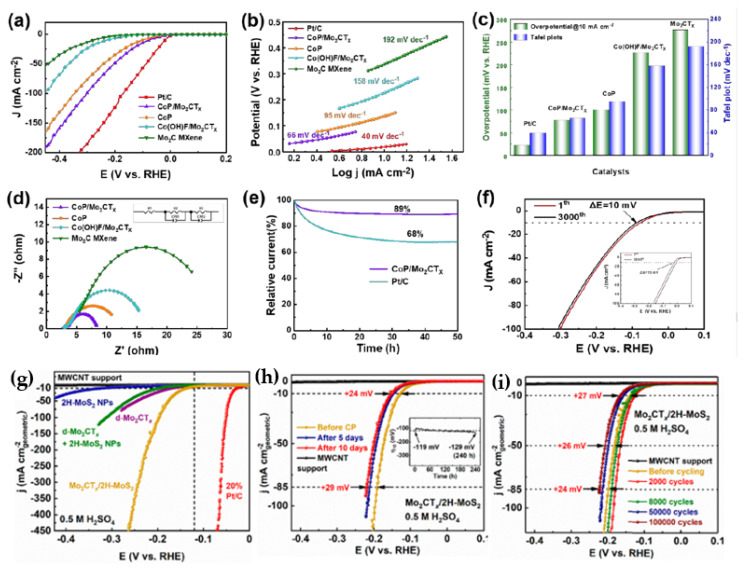
(**a**) LSV curves, (**b**) Tafel plots, (**c**) The Tafel slope and overpotential @10 mA cm^−2^ for Pt/C, CoP/Mo_2_CT_x_, CoP, Co(OH)F/Mo_2_CT_x_, Mo_2_C MXene in 1 M KOH. (**d**) The electrochemical impedance spectra (EIS), (**e**) Chronoamperometric curves of CoP/Mo_2_CT_x_ and Pt/C at the current density of 10 mA cm^−2^ for 50 h. (**f**) LSV curves of CoP/Mo_2_CT_x_ catalyst before and after 3000 CV cycles, and inset is the corresponding LSV curves before and after 3000 CV cycles for Pt/C. Reprinted with permission from Ref. [100]. Copyright 2021, Royal Society of Chemistry. (**g**) iR-corrected CV polarization curves for Mo_2_CT_x_/2H-MoS_2_ nanohybrid in 0.5 M H_2_SO_4_ after (**h**) 10-day CP (constant current at −10 mA cm^−2^_geom_, inset shows time-dependent iR-corrected overpotential during CP) and (**i**) 100,000 accelerated CV cycling at 100 mV s^−1^. HER electrochemical stability results of (Reprinted with permission from Ref. [102] Copyright 2020 American Chemical Society).

Among the other MXenes, the Mo-based MXenes are gaining particular interest due to the fact that the Mo-based-MAX phases can be easily converted into catalytically active 2D MoS_2_. It is well known that, theoretically, 2D MoS_2_ show ideal an ΔG_H_, close to zero. Further synthesis of a solid solution or substitution of cobalt in Mo_2_CT_x_ is found to be one of the effective ways to fine tune the Mo_2_CT_x_ HER activity, especially in alkaline medium. DFT calculations also reveal that the substitution of Co significantly changes the thermodynamics and adsorption energies that favor enhanced HER activity [99]. Liu et al. [100] synthesized CoP-supported Mo_2_CT_x_ for HER reaction. The NH_4_F-etched Mo_2_Ga_2_C produced the layered Mo_2_CT_x_. Mo_2_CT_x_ was negatively charged due to the surface terminations; the Co^2+^ ions were attracted electrostatically. The hydrothermal treatment and subsequent phosphorylation produced the CoP catalyst supported on Mo_2_CT_x_ (CoP/Mo_2_CT_x_). The CoP/Mo_2_CT_x_ showed a HER activity with an overpotential of 78 mV at a current density of 10 mA cm^−2^. Figure 10a shows the LSV curves of CoP/Mo_2_CT_x_^,^, comparing its performance with CoP, Co(OH)F/Mo_2_CT_x_ and Mo_2_C precursor materials. It shows that CoP/Mo_2_CTx had the lowest overpotential at all current densities. It signifies that the synergistic effect between CoP and Mo_2_C MXene promoted the HER catalytic activity compared to the individual materials. Further, to check the reaction kinetics, the Tafel slopes were obtained from the LSV curves, as shown in Figure 12b,c. The obtained Tafel slope values for CoP/Mo_2_CT_x_, CoP, Co(OH)F/Mo_2_CT_x_ and Mo_2_C MXene were 66, 95, 158 and 192 mV dec^−1^, respectively. Among them, CoP/Mo_2_CT_x_ had the lowest Tafel slope, and it was close to the value of Pt/C (40 mV dec^−1^), signifying the identical reaction pathway. The rate determining step was the desorption of H_2_. To test the stability, the CoP/Mo_2_CT_x_ electrode was kept at the current density of 10 mA cm^−2^ for 50 h and observed 89% retention of the initial current, as shown in Figure 12d–f.

Tan et al. [101] developed a mesh carbon-coated MoSe_2_/Mo_2_CT_x_ hybrid material (MoSe_2_/Mo_2_CT_x_@C), which took an overpotential of 108.3 mV to reach 10 mA cm^−2^ current density in acidic medium (0.5 M H_2_SO_4_ solution). The LSV curves of the MoSe_2_/Mo_2_CT_x_@C catalyst are shown along with the individual MoSe_2_ (η = 296.3 mV) and Mo_2_CT_x_ (η = 325.3 mV) catalysts; MoSe_2_/Mo_2_CT_x_@C show good catalytic activity. The obtained Tafel slopes of MoSe_2_/Mo_2_CT_x_@C, MoSe_2_/Mo_2_CT_x_, MoSe_2_ and Mo_2_CT_x_ catalysts were found to be 70.7, 122.1, 108.7 and 152.1 mV dec^−1^, respectively. Among them, MoSe_2_/Mo_2_CTx@C showed the lowest Tafel slope compared to the individual components, and its value showed that it performed the HER via the Volmer–Heyrovsky mechanism. The enhanced reaction kinetics was due to improved conductivity from MoSe_2_, Mo_2_CT_x_ and the carbon coating layer composite. The stability of the MoSe_2_/Mo_2_CT_x_@C catalyst was evaluated using cyclic voltammogram and chronoamperometry techniques. In addition, after 1000 cycles, there was no considerable loss in the activity compared to the initial curve. The chronoamperometric curve of MoSe_2_/Mo_2_CT_x_@C also showed a stable current density up to 24 h, indicating that the good structural stability of MoSe_2_/Mo_2_CT_x_@C was durable. This could be due to the mixed MoSe_2_/Mo_2_CT_x_@C composite. Lim et al. [102] reported Mo_2_CT_x_/2HMoS_2_ nanohybrid catalytic activity in 0.5 M H_2_SO_4_ electrolyte. Due to the intimate epitaxial coupling at the Mo_2_CT_x_/2H-MoS_2_ nanohybrid interface, it afforded superior HER activities, taking only 119 or 182 mV overpotential to reach −10 or −100 mA cm^−2^ current densities, respectively. It is observed that the Tafel slope of Mo_2_CT_x_/2HMoS_2_ was 60 mV dec^−1^, which was significantly lower compared to the bare untreated d Mo_2_CT_x_ (91 mV dec^−1^) and a physical mixture of d-Mo_2_CT_x_ with 2H-MoS_2_ NPs (85 mV dec^−1^). Noticeably, the Mo_2_CT_x_/2H-MoS_2_ nanohybrid’s Tafel slope was maintained up to −450 mA cm^−2^, unlike other metal catalysts, which exhibited increased Tafel slopes, and hence, steeply increasing overpotentials, at higher current densities. In addition, Mo_2_CT_x_/2HMoS_2_ also exhibited excellent stability up to 10,000 cycles (Figure 12g–i).

**Figure 13:**
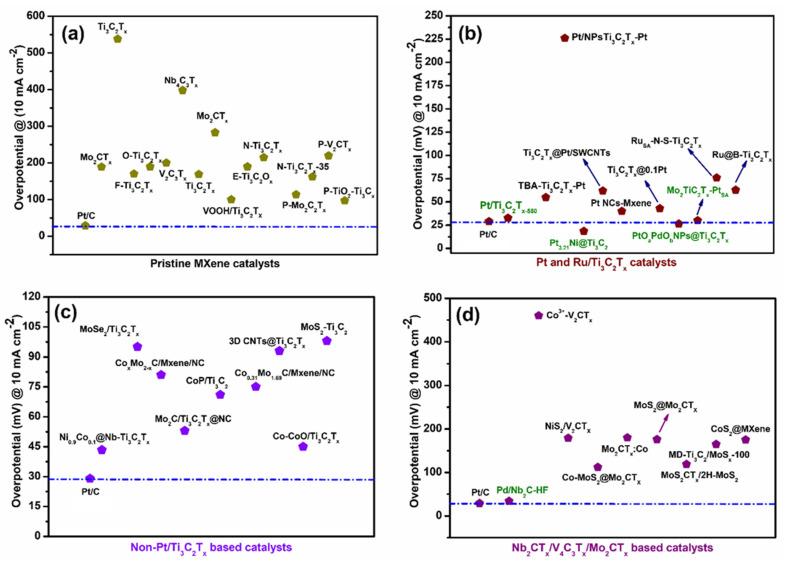
Benchmark figure (**a**) pristine MXene (**b**) Pt-supported Ti_3_C_2_T_x_ (**c**) non-precious metal supported Ti_3_C_2_T_x_ (**d**) Pt and non-precious metal supported on other types of MXenes, other than Ti_3_C_2_T_x_ showing the overpotential status of various MXene-based catalysts with respect to standard Pt/C (for overpotential values of the catalysts, refer to Table 2. The Pt/C reference values were taken from Ref. [102]).

Ren et al. [103] reported 2D organ-like molybdenum carbide (MXene) coupled with MoS_2_ (MoS_2_@Mo_2_CT_x_) nanoflowers. The LSV curves of MoS_2_@Mo_2_CT_x_ were recorded in aqueous 1.0 M KOH solution along with Mo_2_Ga_2_C, Mo_2_CT_x_, MoS_2_ and Pt/C for comparison purpose. The observed overpotential of MoS_2_@Mo_2_CT_x_ was 176 mV at a current density (j) of 10 mA cm^−2^, which was less compared to the values of Mo_2_Ga_2_C (897 mV), Mo_2_CT_x_ (533 mV) and MoS_2_ (394 mV) electrocatalysts. The Tafel slopes of the corresponding as-prepared catalysts were calculated to be 308, 208, 186, 207 and 98 mV dec^−1^ for Mo_2_Ga_2_C, MoS_2_, Mo_2_CT_x_, MoS_2_@Mo_2_CT_x_ and Pt/C, respectively. The smaller Tafel slope of MoS_2_@Mo_2_CT_x_ indicated that the HER may follow the Volmer–Heyrovsky mechanism. The obtained results show that MoS_2_@Mo_2_CT_x_ had a good electrocatalytic activity toward HER, which was due to a greater number of active sites and good electrical conductivity. For useful information to the readers, we summarized the HER performance of MXene catalysts [89,104,105,106,107,108,109,110,111,112,113,114,115,116,117,118,119,120,121,122,123,124,125,126,127,128,129,130,131,132,133,134,135,136,137,138,139,140,141,142] in Table 2. In addition, to compare the status of MXene-based catalysts with the other 2D materials, the HER performances of the catalysts are given in Table 3. After analyzing the HER performance, it was observed that the activities of pristine MXenes were much lower than the other 2D materials. However, the Pt and non-Pt deposited MXenes showed comparable HER performances to other 2D materials, such as graphene, TMDs, LDHs and g-C_3_N_4_-based catalysts, and in some cases, better HER performance. Figure 13 shows the benchmark figures of the Mxene catalysts with respect to commercial Pt/C. The benchmark Figure 13 shows that several MXene-based catalysts showed nearing performance with Pt/C, and some Pt-supported Ti_3_C_2_T_x_ showed an even lower overpotential than Pt/C (indicated by green color in Figure 13). This comparison clearly indicates that with further improvements, MXenes-based catalysts could be potential HER catalyst alternative to traditional Pt/C-based catalysts.

## 7. Conclusions and Future Perspectives

As a “rising star”, MXene-based catalysts have been emerging as potential materials for electrocatalytic hydrogen evolution reaction. The unique 2D structure, chemical, surface and electronic properties provide tremendous opportunities to explore MXens for various electrochemical reactions, including HER. A number of families of MXenes are currently being explored for HER electrocatalysis, which include N-doped MXenes, surface-functionalized MXenes, Ti, V, Nb and Mo based MXenes. Among them, Mo and Ti based MXenes showed better HER activities, which can be further improved by developing effective synthesis strategies, by surface modification, lattice substitution, modulating the defects and controlling the morphology of the supported metal nanoparticles. Among the various surface functional groups (-OH, -O and -F), the -F terminations are found to be important and essential for HER kinetics. Therefore, adjusting the surface functional groups on MXenes might have a direct effect on the HER kinetics. Various Pt and non -Pt metal nanoparticles deposited MXenes were investigated. Among them, Pt-supported MXenes showed excellent HER activity, surpassing the commercial, benchmarking Pt/C catalyst in acidic electrolytes (in a few studies). However, non-Pt-supported MXenes, heteroatom-doped MXenes still showed lower HER kinetics, resulting in high overpotential.

In addition, a number of problems exist in the commercialization aspects of MXene-based electrocatalysts.

1. For example, the high surface energy of 2D MXene leads to re-stacking and agglomeration during catalyst synthesis. Re-stacking reduces the active surface area that needs to be exposed during electrochemical reactions. Therefore, avoiding re-stacking is one of the important factors for obtaining desirable HER activity. The use of intercalating agents, such as large-sized organic molecules, use of ionic surfactants and conducting polymers are some of the strategies.
2. The productivity of the obtained MXenes is highly dependent on the type of synthesis process used, the composition and the number of layers. Further, MXene spontaneous oxidation or thermal-induced structural transformation to TiO_2_ are some of the critical problems that need to be addressed.
3. Increasing MXenes’ intrinsic activity is one of the important issues. Intrinsic activity depends on the type of metal atoms, density of C and N, and the type of surface functional groups and their interactions with the reaction intermediates is vital to obtain better catalysts. For example, -F and -OH functional groups are important for HER and other reactions, such as ORR and OER.
4. One of the major problems associated with MXenes lies in the synthesis process. For example, universally accepted fluoride etching is harmful to the environment and the structure of the MAX phases. The dissolved oxygen present in the aqueous HF can impart structural defects and promote their degradation into TiO_2_.
5. Alternative synthesis strategies, such as electrochemical etching, produce a low yield, and molten salt requires high temperature, making it difficult to industrialize.
6. Careful tuning and reproducible density of surface functional groups are very difficult to achieve due to the fact that the type and density of surface functional groups highly depend on the type of the etchant and reaction conditions.

Despite the excellent progress, MXene-based electrocatalysts are in the nascent stage.

The following strategies are recommended for improved HER kinetics of MXenes.

1. Strategies for controlling surface functional groups, creating porous structures, heteroatom doping, thereby optimizing the electronic and surface properties for selective HER kinetics, should be discovered.
2. Efforts are needed to increase the electronic conductivity of pristine MXenes by producing composite structures with carbon, graphene or by coating of the carbon on MXenes layers, by making 3D architectures that can act as ideal support for dispersing metal nanoparticles and creating interstitial pores for enhancing mass transport issues.
3. It is important to develop 3D porous network structures in MXene and MXenes-composite catalysts to enhance the mass transport properties of the catalysts.
4. While processing the MXenes is also one of the difficult tasks, as the MXenes are sensitive to oxidation, controlled etching, flake structure and heat treatment are required. Treating MXenes at higher temperatures leads to structural transformation of MXenes; for example, Ti_3_C_2_T_x_ converts into TiO_2_ upon heat treatment. Novel synthesis protocols are badly needed for processing MXenes at high temperatures.
5. Environmentally friendly etching processes need to be developed with a high yield and low structural defects. The currently used HF etching is found to be environmentally unfriendly, owing to the acute toxicity of HF.
6. Studies are required to find out if any pre-processing of the MAX phases has any effect on the subsequent etching step.
7. Efforts are needed to understand the effect of surface functionalities (other than -F, -OH and -O), such as -Cl, -Br, -I, -S, -B, -P, -Se, -Te, etc., on the HER activity of MXenes.
8. The durability of MXenes needs to be investigated in long-term operations. Most of the studies performed short-term stability tests, which cannot be guaranteed for long-term cycling operations. DFT calculations explored a number of possible MXenes catalysts as potential candidates for HER, especially the M′_2_M″CNO_2_ type of catalysts. Therefore, it is an interesting task for the experimental scientists to synthesize and study the M′_2_M″CNO_2_ type of MXenes experimentally in order to validate the DFT studies.
9. Advanced strategies, such as lattice substitution of M and X, metal-ion/non-metal ion substitution, defect engineering, morphology control, constructing stable heterojunctions of MXenes with other carbon and conductive 2D materials, are some of the approaches that can be explored.

With increased attention and continuous research evidenced by the rapidly increasing publications on MXenes, there is no doubt that MXene electrocatalysts will gather pace and will be explored for practical HER applications in the near future.

## Figures and Tables

**Table 1:** HER reactions in acidic and alkaline electrolytes and their respective Tafel slopes.

Reaction	Acidic Medium	Basic Medium	Tafel Slop
Volmer step	H^+^ + e^−^ → H*	H_2_O + e^−^ → H* + OH^−^	120 mV dec^−1^
Heyrovsky step	H* + H^+^ + e^−^→ H_2_	H* + H_2_O + e^−^ → OH^−^ + H_2_	40 mV dec^−1^
Tafel step	2H* → H_2_	2H_2_O + 2e^−^ → 2OH^−^ + H_2_	30 mV dec^−1^

**Table 2:** Summary of electrochemical performance of MXene-supported catalysts for HER.

Catalyst	Overpotential (mV) @ 10 mA cm^2^	Electrolyte	Ref
Pristine/Functionalized/Heteroatom-doped MXenes			
Mo_2_CT_x_	189	0.5 M H_2_SO_4_	[104]
Ti_3_C_2_T_x_	538	0.5 M H_2_SO_4_	[105]
F-Ti_2_CT_x_	170	0.5 M H_2_SO_4_	[75]
O-Ti_3_C_2_T_x_	190	0.5 M H_2_SO_4_	[106]
V_4_C_3_T_x_	200	0.5 M H_2_SO_4_	[96]
Nb_4_C_3_T	398	1.0 M KOH	[91]
Ti_3_C_2_T_x_ nanofibers	169	0.5 M H_2_SO_4_	[107]
VOOH/Ti_3_C_2_T_x_	100	1.0 M KOH	[108]
N-Ti_3_C_2_T_x_	215	0.5 M H_2_SO_4_	[77]
N-Ti_3_C_2_T_x_@600	198	0.5 M H_2_SO_4_	[78]
P-Mo_2_CT_x_	114	0.5 M H_2_SO_4_	[109]
N-Ti_3_C_2_T_x_-35	162	0.5 M H_2_SO_4_	[79]
P-V_2_CT_x_	220	0.5 M H_2_SO_4_	[102]
P-TiO_2_@Ti_3_C_2_	97	1.0 M KOH	[110]
Pt/Ti_3_C_2_T_x_-based catalysts			
Pt/C	29	0.5 M H_2_SO_4_	[102]
Pt/Ti_3_C_2_T_x-550_	32.7	1.0 M HClO_4_	[111]
TBA-Ti_3_C_2_T_x_-Pt-20	70	0.5 M H_2_SO_4_	[81]
TBA-Ti_3_C_2_T_x_-Pt	55	0.5 M H_2_SO_4_	[81]
PtNPs/Ti_3_C_2_T_x_	226	0.1 M H_2_SO_4_	[112]
Pt_3.21_Ni@Ti_3_C_2_	18.55	0.5 M H_2_SO_4_	[106]
Pt_3.21_Ni@Ti_3_C_2_	55.6	0.1 M KOH	[113]
Ti_3_C_2_T_x_@Pt/SWCNTs	62	0.5 M H_2_SO_4_	[114]
Pt NCs-MXene	40	0.5 M H_2_SO_4_	[115]
40Pt-TBA-Ti_3_C_2_T_x_	67.8	0.5 M H_2_SO_4_	[80]
Ti_3_C_2_T_x_@0.1Pt	43	0.5 M H_2_SO_4_	[116]
PtO_a_PdO_b_NPs@Ti_3_C_2_T_x_	26.5	0.5 M H_2_SO_4_	[117]
Mo_2_TiC_2_T_x_-Pt_SA_	30	0.5 M H_2_SO_4_	[118]
Ru_SA_-N-S-Ti_3_C_2_T_x_	76	0.5 M H_2_SO_4_	[119]
Ru@B-Ti_3_C_2_T_x_	62.9	0.5 M H_2_SO_4_	[120]
Non-Pt/Ti_3_C_2_T_x_-based catalysts			
Ni_0.9_Co_0.1_@Nb-Ti_3_C_2_T_x_	43.4	1.0 M KOH	[90]
Co^3+^-Ti_2_CT_x_	458	1.0 M KOH	[121]
Ni_0.9_Fe_0.1_PS_3_@ Ti_3_C_2_T_x_	196	1.0 M KOH	[122]
MoS_2_/Ti_3_C_2_-MXene@C	135	0.5 M H_2_SO_4_	[123]
MoS_2_/Ti_3_C_2_T_x_ nanoroll	168	0.5 M H_2_SO_4_	[124]
CoP@3D Ti_3_C_2_T_x_	168	1.0 M KOH	[125]
MoSe_2_/Ti_3_C_2_T_x_	95	1 M KOH	[126]
Co_x_Mo_2-x_C/MXene/NC	75	1.0 M KOH	[127]
Co_x_Mo_2-x_C/MXene/NC	81	0.5 M H_2_SO_4_	[127]
Mo_2_C/Ti_3_C_2_T_x_@NC	53	0.5 M H_2_SO_4_	[128]
Mo_2_C/Ti_3_C_2_T_x_@NC	75	1.0 M KOH	[128]
NiFe-LDH/Ti_3_C_2_T_x_	132	1.0 M KOH	[129]
NiFe-LDH/MXene/NF	205	1.0 M KOH	[129]
P-Mo_2_C/Ti_3_C_2_@NC	177	0.5 M H_2_SO_4_	[130]
CoP/Ti_3_C_2_	71	0.5 M H_2_SO_4_	[131]
CoP/Ti_3_C_2_ MXene	102	1.0 M KOH	[131]
TiOF_2_@Ti_3_C_2_T_x_	103	0.5 M H_2_SO_4_	[132]
Co_0.31_Mo_1.69_C/MXene/NC	75	1.0 M KOH	[133]
Ag@N-Ti_3_C_2_T_x_	153	1.0 M KOH	[134]
3D CNTs@Ti_3_C_2_T_x_	93	1.0 M KOH	[135]
NiSe_2_/Ti_3_C_2_T_x_	200	2.0 M KOH	[89]
Co-CoO/Ti_3_C_2_-MXene	45	1.0 M KOH	[136]
MoS_2_@Ti_3_C_2_	110	0.5 M H_2_SO_4_	[137]
MoS_2_-Ti_3_C_2_	98	0.5 M H_2_SO_4_	[138]
NiFe_2_O_4_/Ti_3_C_2_	173	0.5 M KOH	[139]
Nb_2_CT_x_/ V_4_C_3_T_x_/ Mo_2_CT_x_-based catalysts			
Pd/Nb_2_C-HF	34	0.5 M H_2_SO_4_	[76]
Co^3+^-V_2_CT_x_	460	1.0 M KOH	[122]
NiS_2_/V_2_CT_x_	179	1 M KOH	[140]
Co-MoS_2_@Mo_2_CT_x_	112	1.0 M KOH	[141]
Mo_2_CT_x_:Co	180	1 N H_2_SO_4_	[99]
MoS_2_@Mo_2_CT_x_	176	1.0 M KOH	[103]
MoS_2_CT_x_/2H-MoS_2_	119	0.5 M H_2_SO_4_	[102]
MD-Ti_3_C_2_/MoS_x_-100	165	0.5 M H_2_SO_4_	[142]
NiSe_2_/Ti_3_C_2_T_x_	200	0.5 M H_2_SO_4_	[89]

**Table 3:** Comparison of MXenes HER performance with the other 2D-layered non-precious-based electrocatalysts in acidic and alkaline media.

2D Material	2D Materials-Based Catalysts	Electrolyte	Overpotential (mV) at 10 mA cm^−2^	Ref
Graphene	MoS_2_@pr-GO	0.5 M H_2_SO_4_	263	[143]
	P-doped WN/r-GO	0.5 M H_2_SO_4_	85	[144]
	NG@Co@Zn@NF-850	1 M KOH	34	[145]
	Ni_3_S_2_@NGCLs/NF	1 M KOH	134	[146]
	Porous MoSe_2_ Nanosheets	0.5 M H_2_SO_4_	150	[147]
	ReSe_2_ nanoflakes/rGO	0.5 M H_2_SO_4_	145	[148]
	Ni_0.85_Se nanospheres/rGO	1 M KOH	128	[149]
	Conducting scaffold-supported 3D rGO-CNT/MoS_2_ nanostructure	0.5 M H_2_SO_4_	123 @ 100 mA cm^−2^	[150]
	Conducting scaffold-supported 3D rGO-CNT/MoS_2_ nanostructure	1 M KOH	217 @ 50 mA cm^−2^	[150]
	3D Pd nanosponge-shaped networks wrapped by graphene dots	0.5 M H_2_SO_4_	32	[151]
	3D graphene hollow nanospheres supported ruthenium phosphides	1 M KOH	25.5	[152]
	Ru Nanoclusters/N-graphene	1 M KOH	25.6	[153]
	FeCoNiB@Boron-doped vertically aligned graphene arrays	1 M KOH	31	[154]
	Plasma-etched, S-doped graphene	0.5 M H_2_SO_4_	178	[155]
	3D porous NG derivative integrated MoS_2_ nanosheet	0.5 M H_2_SO_4_	157	[156]
	3D FeP NT/PG	0.5 M H_2_SO_4_	69	[157]
	A self-supporting P-Fe_3_O_4_@3DG bulk composite	1 M KOH	123	[158]
	Ni_2_P nanoparticles/N,B-graphene	1 M KOH	92	[159]
	Dispersed tungsten (W)-optimized MoP nanoparticles on N,P-doped graphene oxide	1 M KOH	70	[160]
	Ni-Ni_3_P@NPC/rGO	0.5 M H_2_SO_4_	113 @ 20 mA cm^−2^	[161]
	Cobalt phosphide decorated/N,B-3D-graphene	0.5 M H_2_SO_4_	118	[162]
TMDs	V-SACs@ 1T-WS_2_	0.5 M H_2_SO_4_	185	[163]
	NiS@ MoS_2_-20	1 M KOH	146	[164]
	MoS_2/_GO	0.5 M H_2_SO_4_	13.1	[165]
	VS_2_@V_2_C	1 M KOH	138	[166]
LDHs	Ni_2_Cr_1_-LDH	1 M KOH	67	[167]
	Co_2_Mn_1_-DH	1 M KOH	187	[168]
	V-Ce/CoFe LDH	1M KOH	73	[169]
g-C_3_N_4_	g-C_3_N_4_/FeS_2_/MoS_2_	0.5 M H_2_SO_4_	193	[170]

## Data Availability

Not applicable.
